# The trans-omics landscape of COVID-19

**DOI:** 10.1038/s41467-021-24482-1

**Published:** 2021-07-27

**Authors:** Peng Wu, Dongsheng Chen, Wencheng Ding, Ping Wu, Hongyan Hou, Yong Bai, Yuwen Zhou, Kezhen Li, Shunian Xiang, Panhong Liu, Jia Ju, Ensong Guo, Jia Liu, Bin Yang, Junpeng Fan, Liang He, Ziyong Sun, Ling Feng, Jian Wang, Tangchun Wu, Hao Wang, Jin Cheng, Hui Xing, Yifan Meng, Yongsheng Li, Yuanliang Zhang, Hongbo Luo, Gang Xie, Xianmei Lan, Ye Tao, Jiafeng Li, Hao Yuan, Kang Huang, Wan Sun, Xiaobo Qian, Zhichao Li, Mingxi Huang, Peiwen Ding, Haoyu Wang, Jiaying Qiu, Feiyue Wang, Shiyou Wang, Jiacheng Zhu, Xiangning Ding, Chaochao Chai, Langchao Liang, Xiaoling Wang, Lihua Luo, Yuzhe Sun, Ying Yang, Zhenkun Zhuang, Tao Li, Lei Tian, Shaoqiao Zhang, Linnan Zhu, Ashley Chang, Lei Chen, Yiquan Wu, Xiaoyan Ma, Fang Chen, Yan Ren, Xun Xu, Siqi Liu, Jian Wang, Huanming Yang, Lin Wang, Chaoyang Sun, Ding Ma, Xin Jin, Gang Chen

**Affiliations:** 1 Cancer Biology Research Center (Key Laboratory of the Ministry of Education), Tongji Medical College, Tongji Hospital, Huazhong University of Science and Technology, Wuhan, China; 2 Department of Gynecologic Oncology, Tongji Hospital, Tongji Medical College, Huazhong University of Science and Technology, Wuhan, China; 3 BGI-Shenzhen, Shenzhen, China; 4 Department of Laboratory Medicine, Tongji Hospital, Tongji Medical College, Huazhong University of Science and Technology, Wuhan, China; 5 BGI Education Center, University of Chinese Academy of Sciences, Shenzhen, China; 6 Department of Gynecology and Obstetrics, Tongji Hospital, Tongji Medical College, Huazhong University of Science & Technology, Wuhan, China; 7 Department of Clinical Laboratory, Union Hospital, Tongji Medical College, Huazhong University of Science and Technology, Wuhan, China; 8 Department of Occupational and Environmental Health, Key Laboratory of Environment and Health, Ministry of Education and State Key Laboratory of Environmental Health (Incubating), School of Public Health, Tongji Medical College, Huazhong University of Science and Technology, Wuhan, China; 9 Department of Research, Xiangyang Central Hospital, Hubei University of Arts and Science, Xiangyang, Hubei, China; 10 Department of Obstetrics and Gynecology, Xiangyang Central Hospital, Hubei University of Arts and Science, Xiangyang, Hubei, China; 11 Department of Gynecologic Oncology, State Key Laboratory of Oncology in South China, Collaborative Innovation Center for Cancer Medicine, Sun Yat-Sen University Cancer Center, Guangzhou, China; 12 Key Laboratory of Tropical Translational Medicine of Ministry of Education, Hainan Medical University, Haikou, China; 13 BGI-Guizhou, BGI-Shenzhen, Guiyang, China; 14 School of Biology and Biological Engineering, South China University of Technology, Guangzhou, China; 15 BGI-Hubei, BGI-Shenzhen, Wuhan, China; 16 College of Veterinary Medicine, Yangzhou University, Yangzhou, China; 17 HIV and AIDS Malignancy Branch, Center for Cancer Research, National Cancer Institute, National Institutes of Health, Bethesda, MD USA; 18 Department of Biochemistry, University of Cambridge, Cambridge, UK; 19 Guangdong Provincial Key Laboratory of Genome Read and Write, BGI-Shenzhen, Shenzhen, China; 20 James D. Watson Institute of Genome Science, Hangzhou, China; 21 School of Medicine, South China University of Technology, Guangzhou, Guangdong, China; 22 Guangdong Provincial Key Laboratory of Human Disease Genomics, Shenzhen Key Laboratory of Genomics, BGI-Shenzhen, Shenzhen, China

**Keywords:** Antimicrobial responses, Gene regulation in immune cells, Viral infection, Systems analysis

## Abstract

The outbreak of coronavirus disease 2019 (COVID-19) is a global health emergency. Various omics results have been reported for COVID-19, but the molecular hallmarks of COVID-19, especially in those patients without comorbidities, have not been fully investigated. Here we collect blood samples from 231 COVID-19 patients, prefiltered to exclude those with selected comorbidities, yet with symptoms ranging from asymptomatic to critically ill. Using integrative analysis of genomic, transcriptomic, proteomic, metabolomic and lipidomic profiles, we report a trans-omics landscape for COVID-19. Our analyses find neutrophils heterogeneity between asymptomatic and critically ill patients. Meanwhile, neutrophils over-activation, arginine depletion and tryptophan metabolites accumulation correlate with T cell dysfunction in critical patients. Our multi-omics data and characterization of peripheral blood from COVID-19 patients may thus help provide clues regarding pathophysiology of and potential therapeutic strategies for COVID-19.

## Abstract (web summary)

COVID-19 is a critical public health threat, but molecular characterizations of patients’ immunity is still lacking. Here the authors collected blood from patients with various disease severity, and prefiltered to exclude selected comorbidity, to obtain genomic, transcriptomic, proteomic, metabolomic and lipidomic profiles to report a trans-omics landscape.

## Introduction

Coronavirus disease 2019 (COVID-19), a newly emerged respiratory disease caused by severe acute respiratory syndrome coronavirus 2 (SARS-CoV-2), was declared a pandemic in early 2020^1^. COVID-19 severity varies dramatically, from asymptomatic to critical. While various studies have reported on patients who exhibit no symptoms^2–5^, the proportion of asymptomatic patients is not precisely known, but appears to range from 13% in children^6^ to 50% during contact tracing^7^. Of those COVID-19 patients with symptoms, 80% are classified as mild to moderate, 13.8% as severe, and 6.2% as critical^1,8^. Certain confounding factors are associated with COVID-19 progress and prognosis. For example, preliminary evidence suggests that comorbidities, such as hypertension, diabetes, cardiovascular disease, and respiratory disease, result in poorer prognosis^9^ and increased mortality^10^. Furthermore, death due to COVID-19 is significantly more common in older patients, which is possibly due to the decline in immune response with age^9,11^. The rate varies from 0.2 to 22.7% depending on the age and health issues of the patient^12,13^.

To date, most studies on COVID-19 have focused on the relationship between disease and clinical characteristics, viral genome sequencing^14^, and identifying the structure of the SARS-CoV-2 spike glycoprotein^15,16^. There has been some work on integrated multi-omics signatures, including meta-transcriptome sequencing of bronchoalveolar lavage fluid^16^ and proteomic and metabolomic analyses of serum from SARS-CoV-2-infected patients^17–19^. However, it remains difficult to determine which parameters are due to viral infection and which to comorbidities as no systematic study of the disease has yet been published.

In the current study, we identified several trans-omics characteristics among patients with different disease severity. We revealed previously unknown and significantly different trans-omics patterns between asymptomatic and symptomatic patients and between critically ill people and other groups. From intensive study of these distinctions, we find that critical patients showed activation of apoptotic processes and phenylalanine and tryptophan (Trp) metabolism. In general, our trans-omics insights into this disease will contribute to our understanding of the underlying pathogenesis of COVID-19 and potential therapeutic strategies.

## Results

### Patient enrollment

To gain insight into the molecular characteristics of COVID-19 patients with different disease severities, a cohort of 231 out of 1432 COVID-19 patients were selected based on stringent criteria for trans-omics study (Supplementary Fig. 1). Given that older age and comorbidities appear to have effects on disease progression and prognosis^20,21^, participants without selected comorbidities and aged between 19 and 70 years old (mean ± SD, 46.7 ± 13.5) were selected. Detailed information on the enrolled patients, including sampling date and basic clinical information, is shown in Supplementary Fig. 2 and Supplementary Data 1–2. Among the enrolled COVID-19 patients, 64 were asymptomatic, 90 were mild, 55 were severe, and 22 were critical.

### Trans-omics profiling for COVID-19

**Fig. 1:**
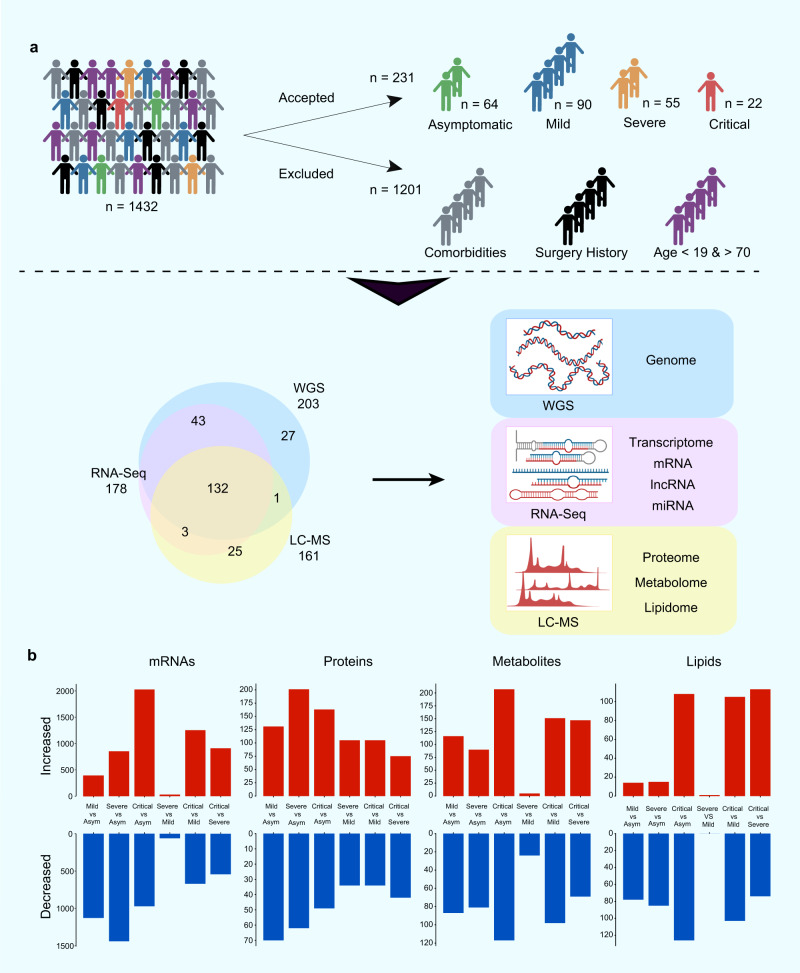
Patient enrollment, study design, and trans-omics profiling of COVID-19. **a** Overview of COVID-19 patient enrollment criteria and study design, including multi-omics profiling of blood samples from COVID-19 patients spanning four disease severities (asymptomatic (asym), mild, severe, and critical). Venn diagram showing overlapping samples profiled using WGS, RNA-seq, and LC-MS. **b** Bar plot showing the numbers of significantly differentially expressed mRNAs, proteins, metabolites, and lipids in six groups of comparison. The mRNA analysis included asymptomatic (*n* = 64), mild (*n* = 64), severe (*n* = 34), and critical (*n* = 16) COVID-19 patients. *P* values for mRNAs were calculated using Wald test (two-sided). The proteins, metabolites, and lipids analysis included asymptomatic (*n* = 53), mild (*n* = 54), severe (*n* = 33), and critical (*n* = 21) COVID-19 patients. *P* values for proteins, lipids and metabolites were calculated using Mann–Whitney U test (two-sided). Multiple comparisons adjustment was performed using Benjamini-Hochberg (BH) method. Exact *P* value and source data were included in the Source Data file.

In-depth multi-omics profiling was performed, including whole-genome sequencing (203 samples) and transcriptome sequencing (RNA-seq and miRNA-seq of 178 samples) of whole blood. Concurrently, liquid chromatography-mass spectrometry (LC-MS) was conducted to capture the proteomic, metabolomic, and lipidomic features of COVID-19 patient serum (161 samples) (Fig. 1a). After data pre-processing and annotation, the final dataset contained 25882 analytes, including 18624 mRNAs, 240 miRNAs, 5207 lncRNAs, 634 proteins, 814 metabolites, and 742 complex lipids (Supplementary Fig. 3, and Supplementary Data 3.1). To quantify molecular profiles in relation to disease severity, we conducted pairwise comparisons between the four disease severity groups for each -omics leve (see Methods). Results indicated extensive changes across all-omics levels (Fig. 1b, Supplementary Fig. 4, and Supplementary Data 3.2–3.5). First, we identified profound differences between asymptomatic and symptomatic patients at all-omics levels. Second, changes in analytes between the mild and severe groups were subtle at all-omics levels, except for proteins. Third, differences between the critically ill group and other groups were extremely high, implying a sudden and dramatic change in critical disease.

### Genomic architecture of COVID-19 patients

After data quality control based on whole-genome sequencing of 203 unrelated patients, 15.3 million bi-allelic single nucleotide polymorphisms (SNPs) were used for single-variant association tests to investigate connections among common variants and diversity of clinical manifestations (Supplementary Fig. 5a–j and Supplementary Data 4.1). We first compared the generalized severe (severe and critical, *n* = 65) and generalized mild groups (asymptomatic and mild, *n* = 138) (Supplementary Fig. 6a, b), then compared asymptomatic (*n* = 63) and all symptomatic patients (*n* = 140) (Supplementary Fig. 6c, d, Supplementary Data 4.2). In general, no signal showed genome-wide significance (*P* < 5e^-8^) in these comparisons. A suggestive signal (*P* < 1e^-6^) associated with the absence of symptoms was found on chromosome 20q13.13, which was comprised of six SNPs, the most significant being rs235001 (Supplementary Data 4.3). Locus zoom identified two protein-coding genes, i.e., *B4GALT5* and *PTGIS*, in the region spanning ±50k of the SNP (Supplementary Fig. 6e). Previous study reported that B4GT5 (encoded by *B4GALT5*) participates in glycosylation of membrane and viral proteins, suggests that it may also play an immunological protection role in porcine respiratory syndrome virus (PRRSV) infection^22^. Together with the *ABO* gene (and glycosyl transferase), altered glycoprotein modification can impact immunogenicity and host immune recognition processes, resulting in differences in susceptibility and severity^23^. *PTGIS* encodes the enzyme for the synthesis of prostaglandin I2, a potent inhibitor of platelet aggregation, thereby inhibiting platelet adherence to vessel walls. In addition, PTGIS possesses anti-inflammatory properties by modulating the expression of interleukin-1 (IL-1), IL-6, and IL-10, which may be associated with COVID-19 severity^24^. We also checked two SNPs, rs657152 at locus 9q34.2 and rs11385942 at locus 3p21.31, which are reportedly associated with severe respiratory failure in Spanish and Italian COVID-19 patients^25^. For rs657152, the overall frequency of protective allele C was 0.5468 (222/406), with the lowest rate found in the critical group (allele frequency (AF) = 0.382, 13/34, Fisher’s exact test *P* = 0.04896). For rs11385942, the risk allele GA was not detected in any patient in our study, as this variant appears to be rare in Chinese population^26^ (Supplementary Data 4.4), consistent with previously reported global distribution^25^.

### Transcriptomic hallmarks of COVID-19

**Fig. 2:**
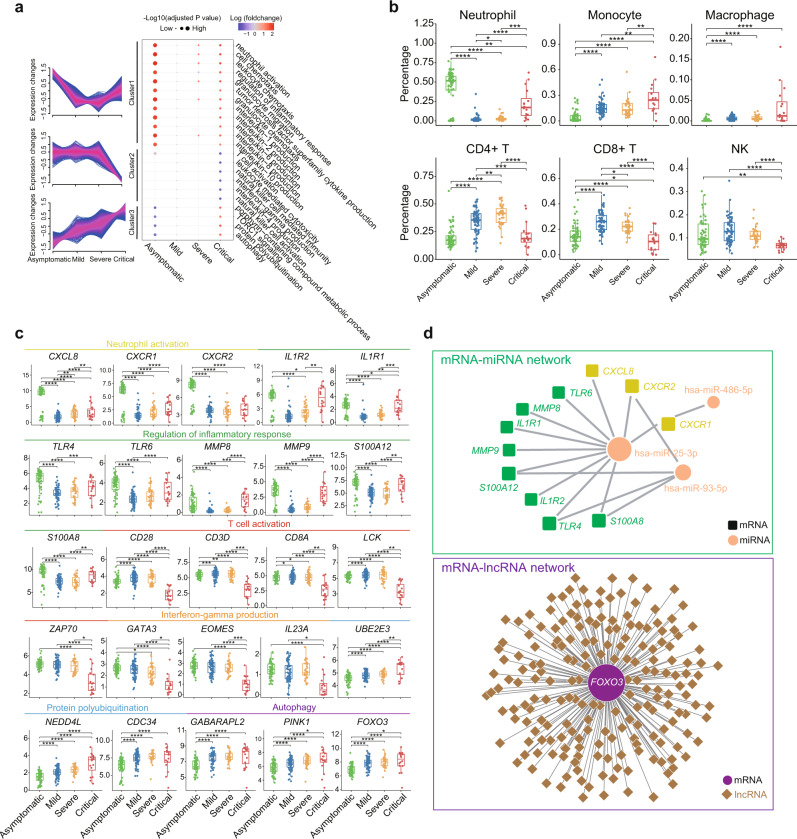
Transcriptomic hallmarks of COVID-19. **a** Clustering of differentially expressed mRNAs and bubble plot showing GO terms enriched in each cluster across four disease severity groups. Red and blue represent up-regulation or down-regulation of genes in corresponding GO term in an investigated group compared to mild group (median of log2 (fold-changes)). Dot size represents -log10 (adjusted P values). P values were calculated using hypergeometric test (two-sided). **b** Estimated immune cell abundance using CIBERSORTx for asymptomatic (*n* = 64), mild (*n* = 64), severe (*n* = 34), and critical (*n* = 16) COVID-19 patients. *P* values were calculated using Wilcoxon signed-rank test (two-sided). **c** Boxplot of representative genes associated with neutrophil activation, regulation of inflammatory response, T cell activation, interferon-gamma production, protein polyubiquitination, and autophagy in asymptomatic (*n* = 64), mild (*n* = 64), severe (*n* = 34), and critical (*n* = 16) COVID-19 patients. We performed comparisons between arbitrary two groups. *P* values were calculated using the Wald test and significant *P* values were shown in boxplot. **d** mRNA-miRNA and mRNA-lncRNA interaction networks for genes mentioned in Fig. 2c. The bold lines, upper boundaries and lower boundaries of notches represent the medians, 75th percentiles and 25th percentiles, respectively. Whiskers extend 1.5 times interquartile range (IQR). * means adjusted *P* value ≤ 0.05, ** means adjusted *P* value ≤ 0.01, *** means adjusted *P* value ≤ 0.001 and **** means *P* ≤ 0.0001, if not indicated, means adjusted *P* value > 0.05. Multiple comparisons adjustment was performed using Benjamini-Hochberg (BH) method. Exact *P* value and source data were included in the Source Data file.

To characterize progressive transcriptional changes in the four disease severity groups, we conducted unsupervised clustering of mRNAs that were differentially expressed (Supplementary Data 3.2). Three expression patterns were identified across patients with different disease severities (Fig. 2a, Supplementary Data 5.1). Intriguingly, genes in cluster 1 increased in both asymptomatic and critically ill patients in comparison to mild and severe patients. The extent of up-regulation was greater in asymptomatic cases. Gene Ontology (GO) analysis showed that these genes are related to neutrophil activation, inflammatory response, granulocyte chemotaxis, and IL2, IL-6, and IL-8 production (Fig. 2a, Supplementary Data 5.2). Consistently, CIBRSORTx digital cytometry^27^, a machine learning method used to estimate cell type abundances from bulk transcriptomes, revealed a dramatic increase in neutrophils in asymptomatic and critically ill patients (Fig. 2b). Key chemokines (*CXCL8*, *CXCR1*, and *CXCR2*) for neutrophil activation and accumulation, as well as inflammatory response genes (*TLR4* and *TLR6*) associated with toll-like receptors and several key inflammatory response genes (*MMP8*, *MMP9*, *S100A12*, *S100A8*), shared this expression pattern (Fig. 2c). This suggests highly activated innate immunity and pro-inflammatory responses in asymptomatic and critically ill patients compared to that in mild and severe patients at the transcriptomic level.

Genes in cluster 2 were enriched in T cell activation, leukocyte-mediated cytotoxicity, natural killer (NK) cell-mediated immunity, and interferon (IFN)-gamma production (Fig. 2a). The expression levels of these genes were specifically decreased in critical patients compared to the other three groups. Important genes for T cell activation, such as *CD28*, *LCK*, and *ZAP70*, as well as key transcription factors for IFN-gamma production (*GATA3*, *EOMES*, and *IL23A*), showed this expression pattern (Fig. 2c). Moreover, digital cytometry estimation revealed lower numbers of T and NK cells in critically ill patients (Fig. 2b). Thus, although innate immune responses were activated in both asymptomatic and critically ill patients, T cell-mediated adaptive immune responses were specifically suppressed in critical COVID-19 patients.

Cluster 3 contained genes primarily involved in protein polyubiquitination and autophagy. The expression of genes in this cluster gradually increased from asymptomatic to mild/severe and then peaked in the critical group (Fig. 2a). An important transcription factor encoding gene for autophagy *FOXO3* also displayed this expression pattern (Fig. 2c). Genes in cluster 3 reflected increasing tissue damage and cell death with disease severity.

We next investigated the post-transcriptional regulatory network associated with genes in Fig. 2c. Results showed that *miR-25-3p*, *miR-486-5p*, and *miR-93-5p* were negatively correlated with 11 genes related to inflammatory response and neutrophil activation (Fig. 2d, Supplementary Data 6). Many lncRNAs were strongly and negatively correlated with *FOXO3*, which plays a critical role in autophagy (Fig. 2d, Supplementary Data 7). The expression of *FOXO3*, a negative regulator of the antiviral response^28^, increased with aggravation of patient condition, suggesting differential accumulation of lncRNAs may play a role in the pathogenesis of critically ill COVID-19 patients (Fig. 2c, d).

### Landscape of proteins, metabolites, and lipids in COVID-19

**Fig. 3:**
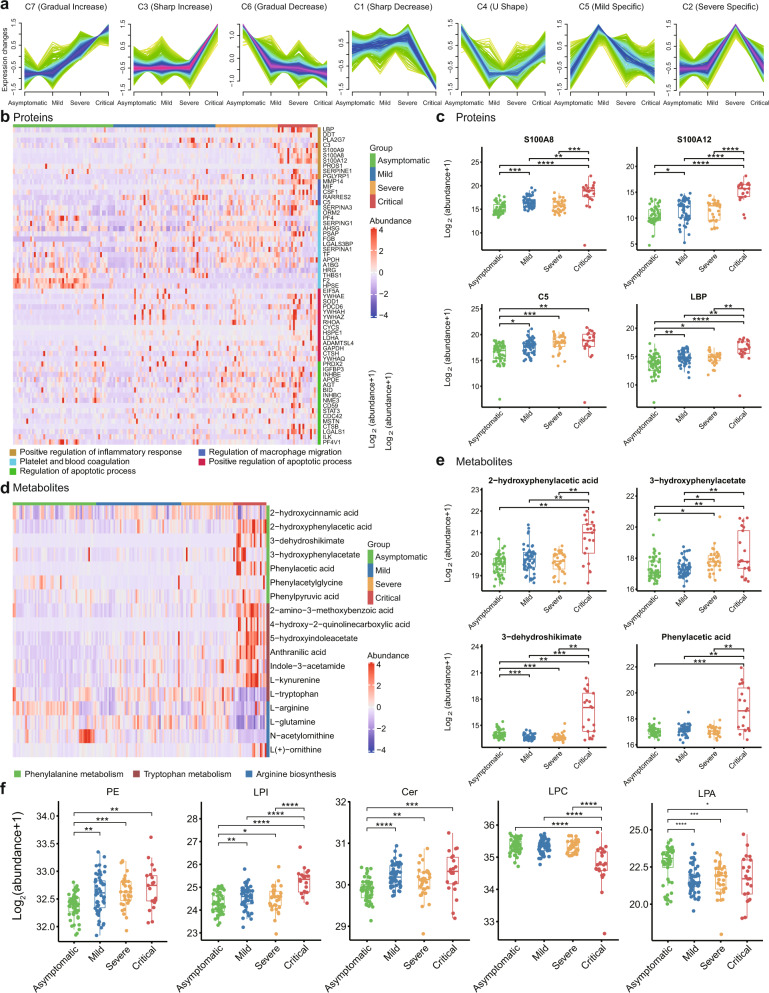
Landscape of proteins, metabolites, and lipids in COVID-19. **a** Expression patterns of COVID-19 plasma analytes, including proteins, metabolites, and lipids, across four disease severity groups. **b** Heatmap representing protein expression in five functional categories in asymptomatic (*n* = 53), mild (*n* = 54), severe (*n* = 33), and critical (*n* = 21) COVID-19 patients. Each column indicates a COVID-19 patient sample, and each row represents a protein. Colors of each cell show Z-score of log2 protein abundance in that sample. **c** Boxplots of representative proteins. We performed comparisons between the arbitrary two groups. The proteins analysis included asymptomatic (*n* = 53), mild (*n* = 54), severe (*n* = 33), and critical (*n* = 21) COVID-19 patients. *P* values were calculated using Wilcoxon signed-rank test (two-sided) and significant *P* values were shown in boxplot. **d** Heatmap representing metabolite expression in phenylalanine, tryptophan metabolism, and arginine biosynthesis pathways in asymptomatic (*n* = 53), mild (*n* = 54), severe (*n* = 33), and critical (*n* = 21) COVID-19 patients. Colors of each cell show Z-score of log2 metabolites abundance in that sample. **e** Boxplots of representative metabolites. We performed comparisons between arbitrary two groups. The metabolites analysis included asymptomatic (*n* = 53), mild (*n* = 54), severe (*n* = 33), and critical (*n* = 21) COVID-19 patients. *P* values were calculated using Wilcoxon signed-rank test (two-sided) and significant *P* values were shown in boxplot. **f** Representative lipid expression changes across four disease severity groups. The lipids analysis included asymptomatic (*n* = 53), mild (*n* = 54), severe (*n* = 33), and critical (*n* = 21) COVID-19 patients. *P* values were calculated using Wilcoxon signed-rank test (two-sided) and significant *P* values were shown in boxplot. The bold lines, upper boundaries and lower boundaries of notches represent the medians, 75th percentiles and 25th percentiles, respectively. Whiskers extend 1.5 times interquartile range (IQR). * means adjusted *P* value ≤ 0.05, ** means adjusted *P* value ≤ 0.01, *** means adjusted *P* value ≤ 0.001 and **** means *P* ≤ 0.0001, if not indicated, means adjusted *P* value > 0.05. Multiple comparisons adjustment was performed using Benjamini-Hochberg (BH) method. Exact *P* value and source data were included in the Source Data file.

Proteins, metabolites, and lipids were classified into seven clusters based on disease severity. Patterns included a gradually increasing cluster (C7), sharply increasing cluster (C3), gradually decreasing cluster (C6), and sharply decreasing cluster (C1). Clusters C4, C5, and C2 showed U-shaped, mild-specific, and severe specific patterns, respectively (Fig. 3a, Supplementary Fig. 7, and Supplementary Data 8).

### Protein circuits in COVID-19

A variety of biological pathways were specifically enriched in patients with different severity (Fig. 3b). Consistent with the transcription analysis (Fig. 2a), several proteins (e.g., LDHA, ADAMTSL4) related to positive regulation of apoptotic processes were preferentially present in critical COVID-19 patients (Fig. 3b). Inconsistent with mRNA expression patterns, proteins associated with positive regulation of the inflammatory response and macrophage migration (S100A8, S100A12, C5, LBP) were gradually or sharply increased (Fig. 3c).

### Metabolite profiles in COVID-19

Multiple metabolic pathways showed distinct profiles in patients with different severity (Fig. 3d). Of note, phenylalanine and Trp metabolism increased sharply in critical patients (Fig. 3d, e). Trp metabolism is considered a biomarker and therapeutic target of inflammation^29^ and changes in Trp metabolism are correlated with serum IL-6 levels^30^. Consistently, IL-6 levels were highest in critical patients (Supplementary Data 2). Results also showed that arginine gradually decreased with disease severity (Fig. 3d). Arginine is metabolized by myeloid cells (neutrophils, macrophages, granulocytes) and arginase^31^, further supporting the activation of neutrophils and macrophages in symptomatic, especially critical, patients.

### “Lipid codes” in COVID-19

We investigated the dynamics of lipids within the different disease severity groups. Phosphatidylethanolamine (PE), lysophosphatidylinositol (LPI), and ceramides (Cer) all gradually increased (Fig. 3f). RNA virus replication is reported to be dependent on enrichment of PE distributed at the replication sites of subcellular membranes^32^. LPI is an endogenous agonist for GPR55, whose activation regulates several pro-inflammatory cytokines^33^. Ceramide induction inhibits T cell cytoskeletal reorganization in measles virus immunosuppression^34^ and may increase the efficiency of pathogen uptake into dendritic cells^35^.

Lysophosphatidylcholine (LPC), whose down-regulation is a strong predictor for sepsis-related mortality^36,37^, was sharply decreased in critical patients (Fig. 3f). Intriguingly, Lysophosphatidic acid (LPA), which can enhance the secretion of IFN-γ by activation of NK cells^38^, was significantly enriched in asymptotic COVID-19 patients (Fig. 3f). Overall, our study suggests that lipidomic changes may play important and complex roles in COVID-19 disease development.

### Distinct neutrophil status within asymptomatic and critically ill COVID-19 patients

**Fig. 4:**
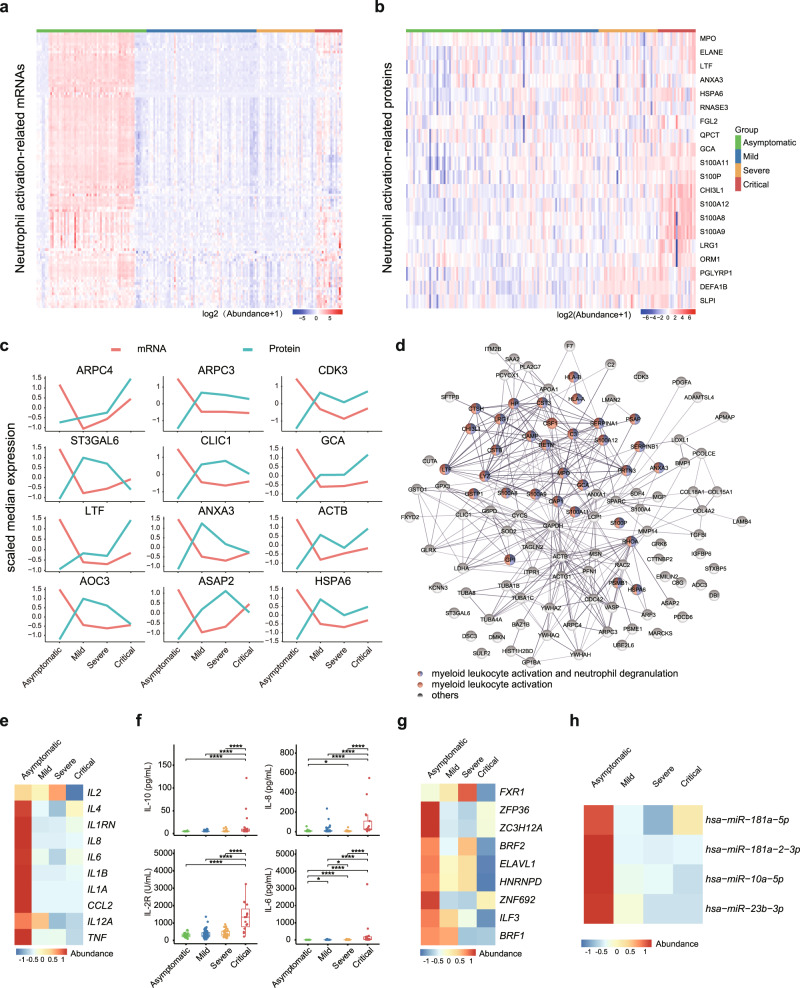
Distinct neutrophil status and “cytokine paradox” within asymptomatic and critically ill COVID-19 patients. **a** Heatmap of mRNA abundance for genes in neutrophil activation pathway in asymptomatic (*n* = 64), mild (*n* = 64), severe (*n* = 34), and critical (*n* = 16) COVID-19 patients. **b** Heatmap of protein abundance in neutrophil activation pathway in asymptomatic (*n* = 53), mild (*n* = 54), severe (*n* = 33), and critical (*n* = 21) COVID-19 patients. **c** Dynamic changes in representative genes showing discordance between protein and mRNA expression. mRNA and protein abundance were scaled by median expression. **d** Protein-protein interaction network (PPIN) of genes showing discrepancy pattern in mRNA and protein abundance. **e** Variation patterns of gene expression of inflammatory cytokines across four disease severity. **f** Quantification of IL-6 (pg/mL), IL-8 (pg/mL), IL-10 (pg/mL), and IL-2R (U/L) in each group. We performed comparisons between arbitrary two groups. *P* values were calculated using Wilcoxon signed-rank test (two-sided) and significant *P* values were shown in boxplot. **g** Heatmap showing mRNA abundance of RBPs across four disease severity groups. **h** Heatmap showing miRNA abundance across four disease severity groups. The bold lines, upper boundaries and lower boundaries of notches represent the medians, 75th percentiles and 25th percentiles, respectively. Whiskers extend 1.5 times interquartile range (IQR). * means adjusted *P* value ≤ 0.05, ** means adjusted *P* value ≤ 0.01, *** means adjusted *P* value ≤ 0.001 and **** means *P* ≤ 0.0001, if not indicated, means adjusted *P* value > 0.05. Multiple comparisons adjustment was performed using Benjamini-Hochberg (BH) method. Exact *P* value and source data were included in the Source Data file.

Neutrophils are the first responders of immune defense and play critical roles in many airway infections, including antiviral immunity^39^. However, excessive neutrophil activation may abnormally differentiate into pathological low-density neutrophils with an enhanced capacity to release neutrophil extracellular traps (NETs)^40^. Excessive NETs release causes endothelial damage, promotes thrombosis, and contributes to mortality in COVID-19^41^. As seen in Fig. 2a, transcriptional analysis showed that neutrophils were massively enriched in asymptomatic patients and mildly increased in critically ill patients (Fig. 4a). However, most proteins (20 detectable in proteomics data), including those involved in inflammatory pathways (CHI3L1, S100A8, S100A9, S100A11, and S100A12), neutrophil degranulation (ANXA3, FGL2, LRG1, PGLYRP1, DEFA1B, and SLPI), and NETs (MPO and ELANE), were extremely low in asymptomatic patients, and progressively increased with disease severity (Fig. 4b). This discrepancy implies that heterogeneous neutrophils, which are “beneficial” or “detrimental”, exist between asymptomatic and critically ill patients. We also analyzed expression correlations with available paired mRNAs and proteins. In total, 111 genes showed the highest transcripts but lowest protein levels in asymptomatic patients (Fig. 4c, d). Remarkably, myeloid leukocyte activation and neutrophil degranulation pathways were enriched in these genes (Fig. 4d), further supporting the notable neutrophil heterogeneity among different disease severity groups.

### “Cytokine paradox” in asymptomatic COVID-19 patients

Pro-inflammatory pathway and inflammatory cytokines were unexpected transcriptionally activated in asymptomatic patients (Figs. 2a and 4e). However, consistent with recent research^42^, secretion of inflammatory cytokines, such as IL-6, IL-8, IL-2R, and IL-10, was extremely low in the serum of asymptomatic patients (Fig. 4f). In contrast, critically ill patients were characterized with excessive inflammatory cytokine production, but only modestly elevated transcription levels (Fig. 4e, f). Typically, inflammatory cytokine production is tightly regulated both transcriptionally and post-transcriptionally^43,44^. Post-transcription of inflammation-related mRNAs is mainly regulated by RNA-binding proteins (RBPs) and microRNAs. Interestingly, RBPs (HNRNPD, ZFP36, ZC3H12A, ILF3, ZNF692, FXR1, ELAVL1, and BRF1/2) and microRNAs (*miR-181a-5p*, *miR-181a-2-3p*, *miR-10a-5p*, *miR-23b-3p*), which might involved in the degradation and destabilization of inflammatory cytokines^45–48^, were highly expressed in asymptomatic patients but lowly expressed in critical patients (Fig. 4g, h).

### Tryptophan and arginine metabolism perturbations, and T cell dysfunction in critically ill COVID-19 patients

**Fig. 5:**
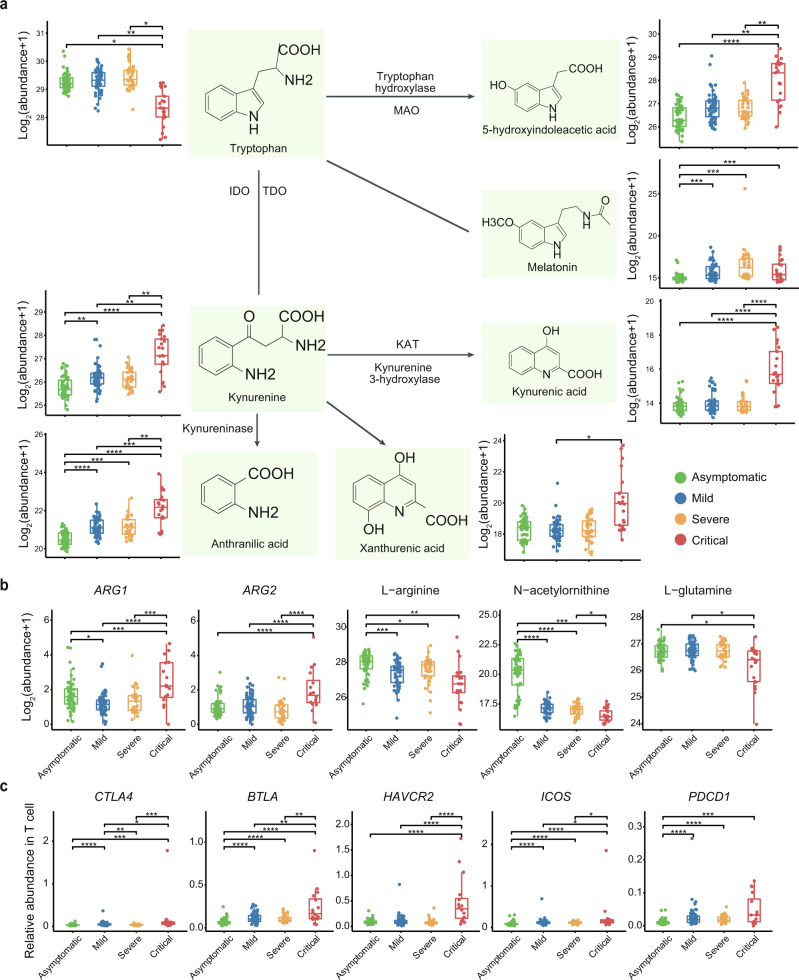
Tryptophan and arginine metabolism perturbations, and T cell dysfunction in critically ill COVID-19 patients. **a** Summary of tryptophan metabolism pathways in asymptomatic (*n* = 53), mild (*n* = 54), severe (*n* = 33), and critical (*n* = 21) COVID-19 patients (IDO, indoleamine 2,3-dioxygenase; KAT, kynurenine aminotransferase; MAO, monoamine oxidase; TDO, tryptophan 2,3-dioxygenase). Box plots show expression level change (log2(x + 1)) of selected regulated metabolites across four disease severities. We performed comparisons between arbitrary two groups. *P* values were calculated using Mann–Whitney U test (two-sided) and significant *P* values were shown in boxplot. **b** Boxplots of mRNA for (*ARG1*, *ARG2*) and metabolic abundance of arginine metabolism pathway components (L-arginine, N-acetylornithine, L-glutamine). We performed comparisons between arbitrary two groups. The mRNA analysis included asymptomatic (*n* = 64), mild (*n* = 64), severe (*n* = 34), and critical (*n* = 16) COVID-19 patients. *P* values were calculated using Wald test (two-sided) and significant *P* values were shown in boxplot. The metabolites analysis included asymptomatic (*n* = 53), mild (*n* = 54), severe (*n* = 33), and critical (*n* = 21) COVID-19 patients. *P* values were calculated using Mann–Whitney U test (two-sided) and significant *P* values were shown in boxplot. **c** Relative expression abundance of exhaustion marker genes *CTLA4*, *BTLA*, *HAVCR2*, *ICOS*, and *PDCD1* in T cells in asymptomatic (*n* = 64), mild (*n* = 64), severe (*n* = 34), and critical (*n* = 16) COVID-19 patients. Relative expression abundance of exhaustion marker genes was defined as their expression levels divided by expression level of T cell marker gene *CD3E*. We performed comparisons between arbitrary two groups. *P* values were calculated using Wald test (two-sided) and significant *P* values were shown in boxplot. The bold lines, upper boundaries and lower boundaries of notches represent the medians, 75th percentiles and 25th percentiles, respectively. Whiskers extend 1.5 times interquartile range (IQR). * means adjusted *P* value ≤ 0.05, ** means adjusted *P* value ≤ 0.01, *** means adjusted *P* value ≤ 0.001 and **** means *P* ≤ 0.0001, if not indicated, means adjusted *P* value > 0.05. Multiple comparisons adjustment was performed using Benjamini-Hochberg (BH) method. Exact *P* value and source data were included in the Source Data file.

T cells play a critical role in antiviral immunity against SARS-CoV-2^49^, but their functional state and contribution to COVID-19 severity remain largely unknown. In critically ill patients, the T/NK cell-mediated adaptive immune response was defective (Fig. 2a, b). Interestingly, Trp metabolism gradually increased with disease severity (Fig. 5a). Trp degradation products deplete T cells, increase T helper (Th) cell and NK cell apoptosis, and promote T cell exhaustion^50^. L-arginine is important for T cell proliferation and function, and the release of arginase (*ARG1/2*) from activated neutrophils inhibits T cell activation by inducing L-arginine and glutamine depletion^51^. Here, we found that *ARG1* and *ARG2* levels were up-regulated in critical patients. Consistently, L-arginine, N-acetylornithine, and L-glutamine were decreased in critical patients (Fig. 5b). Phenotypically, in addition to the dramatic decrease in T cells in critically ill patients (Fig. 2b), exhaustion markers, e.g., *CTLA4*, *BTLA, HAVCR2, ICOS*, and *PDCD1* were significantly up-regulated in critical patients (Fig. 5c).

### Impaired IFN response in critically ill COVID-19 patients

**Fig. 6:**
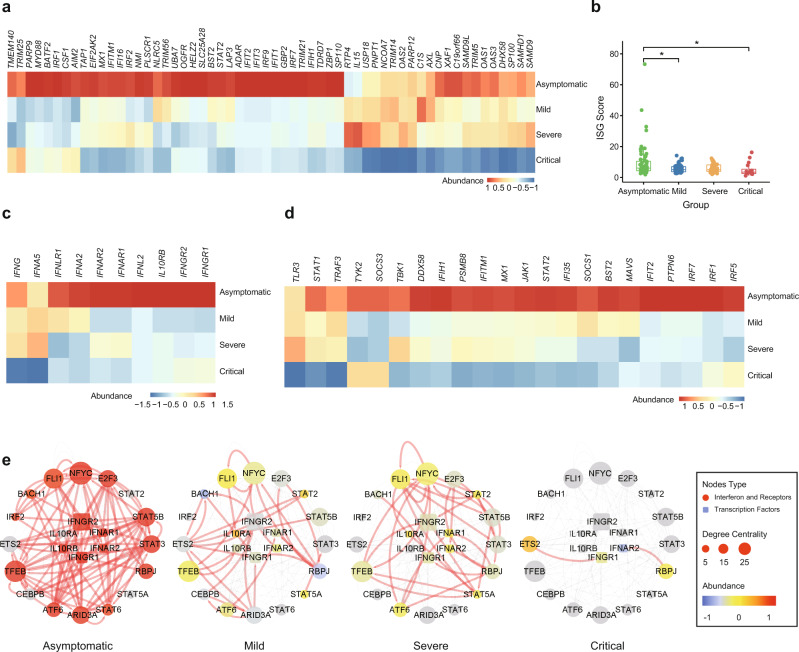
Impaired interferon (IFN) response in critically ill COVID-19 patients. **a** Heatmap demonstrating expression levels of IFN-stimulated genes (ISG) in asymptomatic (*n* = 64), mild (*n* = 64), severe (*n* = 34), and critical (*n* = 16) COVID-19 patients. **b** Quantification of ISG scores (measured by the mean expression of genes mentioned in Fig. 6a) in asymptomatic (*n* = 64), mild (*n* = 64), severe (*n* = 34), and critical (*n* = 16) COVID-19 patients. We performed comparisons between arbitrary two groups. *P* values were calculated using Wald test (two-sided) and significant *P* values were shown in boxplot. The bold lines, upper boundaries and lower boundaries of notches represent the medians, 75th percentiles and 25th percentiles, respectively. Whiskers extend 1.5 times interquartile range (IQR). * means adjusted *P* value ≤ 0.05, ** means adjusted *P* value ≤ 0.01, *** means adjusted *P* value ≤ 0.001 and **** means *P* ≤ 0.0001, if not indicated, means adjusted *P* value > 0.05. Multiple comparisons adjustment was performed using Benjamini-Hochberg (BH) method. Exact *P* value and source data were included in the Source Data file. **c** Heatmap of mRNA of IFN and IFN receptors in asymptomatic (*n* = 64), mild (*n* = 64), severe (*n* = 34), and critical (*n* = 16) COVID-19 patients. **d** Heatmap of mRNA of upstream regulators of IFN signaling in asymptomatic (*n* = 64), mild (*n* = 64), severe (*n* = 34), and critical (*n* = 16) COVID-19 patients. **e** Gene regulatory sub-network of IFN and IFN receptors. Nodes were colored based on mRNA expression abundance, which was scaled in different groups, and size of nodes corresponds to degree centrality. Color and size of edges represent whether a regulation relationship for each pair exists in different groups.

An effective IFN response can eliminate viral infection, including that of SARS-CoV-2^52^. Insufficient activation of IFN signaling may contribute to severe cases of COVID-19^53,54^. As such, we compared the pathways of antiviral IFN responses in the different COVID-19 patient groups. Intriguingly, we found that critically ill patients failed to launch a robust IFN response, as measured by the expression of IFN-stimulated genes (ISGs) (Fig. 6a, b). Furthermore, IFN receptors were much lower in symptomatic patients (Fig. 6c). Multiple IFN upstream molecules, including *TLR3*, *IRF1*, *IRF7*, *MAVS*, *DDX58*, *TBK1*, *JAK1*, and *STAT2*, were down-regulated in symptomatic, especially critical, patients (Fig. 6d). We also performed reverse engineering of the gene regulatory network (GRN) to explore the transcriptional regulation network of the IFN pathway in patients with different disease severity (Supplementary Data 9). In asymptomatic patients, transcription factors, including STAT5B, STAT3, STAT6, E2F3, NFYC, FLI1, ATF6, TFEB, and ARID3A, were strongly connected with IFN or IFN receptors (Fig. 6e). Given the nature of the GRN, a decrease in edge counts indicates a reduced regulatory relationship among genes. A gradual loss of connectivity in the IFN regulatory network was observed in symptomatic groups, especially in critical patients (Fig. 6e), which may contribute to the dysregulation of the IFN pathway in critically ill patients.

## Discussion

To the best of our knowledge, this is the first systematic analysis of trans-omics data from COVID-19 patients with different clinical severity. Through comprehensive multi-omics analysis, we revealed high neutrophil counts and enhanced IFN antiviral response in asymptomatic patients compared with symptomatic patients. In contrast, critically ill patients were characterized by neutrophil over-activation, cytokine storm, and IFN-mediated innate immunity or T-mediated adaptive immunity deficiency.

Asymptomatic patients, as silent spreaders of disease, have garnered considerable attention due to the difficulty in their identification during epidemic control^42^. Unexpectedly, compared with symptomatic COVID-19 patients, asymptomatic patients exhibited expression discordance, with transcriptional activation but low inflammatory cytokine secretion. Typically, inflammatory cytokine production could be regulated both transcriptionally and post-transcriptionally^55,56^. By recognizing those with stem-loop structures, RBPs can degrade or decay inflammatory cytokine mRNAs, and microRNAs have also emerged as fine-tune regulators of inflammation^57^. The balance of these actions controls inflammation intensity^45^. For instance, AUF1 (HNRNPD) and TTP (ZFP36) attenuate inflammation by destabilizing mRNA-encoding inflammatory cytokines, including IL-6, tumor necrosis factor (TNF)^58–60^. Regnase-1 (ZCH12A), which has a broad antiviral spectrum and efficiently inhibits the influenza A virus, can inhibit inflammation by negatively regulating IL6 and IL17 mRNA stabilization^61,62^. MiR-10a and miR-21 negatively regulate IL-6 and TNF^63^. Thus, we propose that the observed discrepancy between cytokine mRNA and protein levels could be associated with post-transcriptional dysregulation. However, additional functional studies are required to ascertain their contribution to the development of COVID-19.

Neutrophils play a protective role in antiviral immunity, with depletion leading to viral replication and increased lethality in mice infected with influenza^64^. Neutrophils exhibit a strong ability to mediate virus elimination, not only by direct phagocytic activity but also in cooperation with B cells, by modulation of dendritic cell (DC), macrophage, and T cell activities^65^. However, excessive neutrophil activation can cause tissue damage^66,67^. Interestingly, Wilk et al. found a novel neutrophil population only specific increased in acute respiratory distress syndrome (ARDS) COVID-19 patients, which supports the heterogeneous neutrophils in different disease severities^68^. We found excessive activation of neutrophils in critically ill COVID-19 patients, although whether these cells were derived from developing neutrophils needs further investigation. NETs are reported to be closely related to influenza, Ebola, and COVID-19 severity^41,69^. These observations hint at a prominent role of neutrophils in COVID-19. Thus, the determinants of neutrophil transition from beneficial to detrimental deserve further investigation. In addition, considering the major role excess NETs play in COVID-19 severity, targeting NET formation by inhibiting molecules critical for their production (e.g., neutrophil elastase (NE), PAD4, and gasdermin D)^70–72^ is a promising therapeutic choice for reducing clinical severity in COVID-19.

T cell depletion in critically ill COVID-19 patients accords with the clinical observation of T cell lymphopenia^73^. Recent research has demonstrated that SARS-CoV-2 infection dramatically reduces T cells and up-regulates exhaustion markers PD-1 and Tim-3, especially in critically ill patients^73^. Mechanistically, clinical evidence shows that T cell counts are negatively associated with serum IL-6, IL-10, and TNF-α concentrations^73^, and uncontrolled cytokine release may prompt the depletion and exhaustion of T cells^74^. Accelerated Trp metabolism by rate-limiting enzymes, i.e., indoleamine 2,3-dioxygenases (IDO1 and IDO2), mediates T cell dysfunction^75,76^. Trp catabolite production, kynurenine (KYN), 3-HAA, and Quin inhibits adaptive T cell immunity, blocks expansion and proliferation of conventional CD4^+^ helper T cells and effector CD8^+^ T cells and potentiates CD4^+^ regulatory T (Treg) cell function^77^. In addition, L-arginine depletion due to hyper-activated neutrophils inhibited T-cell function^78^. Thus, in addition to their reduction in critical patients, T cells also become metabolically exhausted and dysfunctional.

Consistent with our findings, Arunachalam et al. revealed several common immune response features induced upon SARS-CoV-2 infection, such as impaired type I IFN response in the periphery, defective innate response in blood leukocytes, and enhanced inflammatory cytokine S100A12 expression in myeloid cells^79^. The impaired IFN response in critical patients could be responsible for the loss of viral replication control^80^. Uncontrolled viral replication can result in the orchestration of a much stronger inflammatory response in critically ill patients, characterized by cytokine storm and immunopathogenesis^80^.

It is possible that biological crosstalk exists among cytokine storm, Trp metabolism, and T cell dysfunction. First, considering the essential role of Trp metabolism in blocking the expansion and proliferation of conventional CD4^+^ helper T cells and effector CD8^+^ T cells and in potentiating CD4^+^ regulatory T (Treg) cell function^77^, the increase in Trp catabolite production could inhibit adaptive T cell immunity. Second, Trp directly stimulates immune checkpoint expression levels, such as CTLA4 and PD-1^81^. Third, in addition to the direct effects on T cell dysfunction, proinflammatory cytokines, e.g., IL-1β, IFN-γ, and IL-6, can lead to a robust elevation in circulating KYN levels by up-regulation of IDO/TDO^82^, which synergistically worsen T cell dysfunction. Fourth, adaptive T cell immunity plays an unexpected role in tempering the initial innate response^83^, and T cell defection in critically ill patients could exacerbate an uncontrolled innate immune response.

Therapeutically, considering the essential effects of arginine, tryptophan, IDO, and T cell function on COVID-19 severity, bolstering the immune system by restoring exhausted T cells may be a promising strategy for disease treatment. Direct arginine supplementation, targeting Trp catabolism by indoximod, or targeting IDO1/TDO2 by navoximod (NLG919)^84^, BMS-986205^85^, or PF-06840003^86^ could metabolically restore T cell function. Furthermore, immune checkpoint blockage with PD1/PD-L1 or CTLA4 antibodies, which can increase T cell number and restore T cell function^87^, may be a potential strategy for the treatment of critically ill patients. Therefore, it would be worthwhile to test whether these immune-boosting strategies are effective in clinical COVID-19 trials.

We note several limitations with our analysis. First, we did not enroll a healthy population as a control group, so conclusions made in this study are limited to differences in COVID patients with different disease severity. Various stages of COVID-19 were included in our study design to identify key clues or biomarkers to distinguish disease severity and help prevent disease progression. Second, due to strict inclusion criteria, the number of patients with severe or critical disease was relatively small, which may have an impact on our results.

In conclusion, our study presented a trans-omics landscape of blood samples within a large cohort of COVID-19 patients with different disease severity, from asymptomatic to critically ill. Overall, this study provides novel insights and therapeutic targets relevant to COVID-19, as well as valuable clues for deciphering COVID-19 and its underlying mechanisms.

## Methods

### Ethics statement

This study was reviewed and approved by the Institutional Review Board of Tongji Hospital, Tongji Medical College, Huazhong University of Science and Technology, China (TJ-IRB20200405). All enrolled patients provided signed informed consent and all blood samples were collected for the rest of the standard diagnostic tests, with no additional burden to the patients.

### Patient enrollment and sample preparation

Blood samples from 231 COVID-19 patients without selected comorbidities were collected from Tongji Hospital and Union Hospital of Huazhong University of Science and Technology, Xiangyang Central Hospital, Hubei University of Arts and Science, and Hubei Dazhong Hospital of Chinese Traditional Medicine between 19 February and 26 April 2020. A patient selection flowchart is shown in Supplementary Fig. 1. The exclusion criteria of comorbidities included hypertension, coronary heart disease, diabetes, chronic obstructive pulmonary disease, malignancy, surgical history, chronic kidney disease, cerebrovascular disease, immunodeficiency disease, chronic hepatitis, and tuberculosis. Demographic data and laboratory indicators are shown in Supplementary Data 1-2. The mean age of patients was 46.7 years old (standard deviation (SD) = 13.5), and the male to female ratio was 1.12:1. All patients were diagnosed following the guidelines for COVID-19 diagnosis and treatment (Trial Version 7) released by the National Health Commission of the People’s Republic of China based on the course of illness^79^. The patients were classified into four groups according to disease severity: i.e., critical, severe, mild, and asymptomatic. Critical disease was defined with at least one of the following conditions: (1) ARDS requiring mechanical ventilation, (2) shock, and (3) other organ failure requiring ICU admission. Severe disease was defined with at least one of the following conditions: (1) respiratory rate ≥ 30 times/min, (2) oxygen saturation ≤ 93% at resting state, (3) arterial partial pressure of oxygen (PaO_2_)/fraction of inspired oxygen (FiO_2_) ≤300 mmHg, (4) pulmonary imaging showing significant progression of lesions by more than 50% within 24–48 h. Mild disease was defined as patients with mild clinical symptoms but not reaching the definition of severe disease. Asymptomatic disease was defined as patients with normal body temperature and without any respiratory symptoms. The ethylenediaminetetraacetic acid disodium salt (EDTA-2Na)-anticoagulated venous blood samples were separated by centrifugation at 3000 rpm for 7 min at room temperature after standard diagnostic tests. Whole blood cells were stored at −80 °C. A 200-μL aliquot of serum was added to 800 μL of ice-cold methanol, mixed well, and stored at −80 °C. Another 200-μL aliquot of serum was added to 800 μL of ice-cold isopropanol, mixed well, and stored at −80 °C.

### Nucleic acid extraction

A 200-μL aliquot of each thawed whole blood cell sample was used to extract DNA with a QIAamp DNA Blood Mini Kit (51304, Qiagen) following the manufacturer’s instructions. Total RNA was extracted from another 200-μL aliquot of blood cells using a Qiagen miRNeasy Mini Kit (217004, Qiagen) according to the manufacturer’s protocols. All extraction procedures were performed under level III protection in a biosafety III laboratory.

### Sequencing library construction and data generation

Whole-genome data were generated as follows: (1) DNA was randomly fragmented by Covaris. The fragmented genomic DNA was then selected by magnetic beads to an average size of 200–400 bp. (2) Fragments were end repaired and then 3ʹ adenylated. Adaptors were ligated to the ends of these 3ʹ adenylated fragments. (3) PCR and circularization were performed. (4) After library construction and sample quality control, whole-genome sequencing was conducted on the MGI2000 PE100 platform with 100-bp paired-end reads.

Transcriptome RNA data were generated as follows: (1) rRNA was removed using the RNase H method. (2) A QAIseq FastSelect RNA Removal Kit was used to remove globin RNA. (3) Purified fragmented cDNA was combined with End Repair Mix and A-Tailing Mix, then mixed well by pipetting and incubated. (4) PCR amplification was performed. (5) Library quality control and pooling cyclization were conducted. (6) The RNA library was sequenced using the MGI2000 PE100 platform with 100-bp paired-end reads.

Small RNA data were generated as follows: (1) Small RNA was enriched and purified. (2) Adaptor ligation and unique molecular identifier (UMI)-labeled primers were added. (3) RT-PCR, library quantitation, and pooling cyclization were performed. (4) Library quality control was conducted. (5) Small RNAs were sequenced using the BGI500 platform with 50-bp single-end reads, resulting in at least 20 M reads for each sample.

### Cytokine detection

We detected cytokines, including IL-6, IL-8, IL-10, and IL-2R, in patient serum samples. Assays were conducted using an automated analyzer (Cobas e602, Roche Diagnostics, Germany or Immulite 1000, DiaSorin Liaison, Italy) as described in the manufacturers’ instructions. The IL-6 kit (#05109442190) was obtained from Roche Diagnostics (Mannheim, Germany) and the IL-8 kit (#LK8P1), IL-10 kit (#LKXP1), and IL-2R kit (#LKIP1) were obtained from DiaSorin (Vercelli, Italy).

### Whole-genome sequencing data analysis and joint variant calling

Whole-genome sequencing data were processed using Sentieon Genomics (v: sentieon-genomics-201911)^88^. The pipeline was built according to best practice workflows for germline short variant discovery described in https://gatk.broadinstitute.org/. Sequencing reads were mapped to the hg38 reference genome using the BWA algorithm^89^. After duplicate marking, Indel realignment, and base quality score recalibration (BQSR), per-sample variants were called using the Haplotyper algorithm in GVCF mode. The GVCFtyper algorithm was then used to perform joint-calling and generate cohort VCF. Variant quality score recalibration was performed using the Genome Analysis Toolkit (GATK v4.1.2)^90^. The truth-sensitivity-filter-level was set to 99.0 for both SNPs and Indels. Finally, variants with a PASS flag and quality score ≥ 100 were selected for further analysis.

### Genotype-phenotype association analysis

Principal component analysis (PCA) was performed using PLINK (v1.9)^91^. Bi-allelic SNPs were selected based on the following criteria: MAF ≥ 5%; genotyping rate ≥90%; LD prune (window = 50, step = 5 and r2 ≥ 0.5). A subset of 605867 SNPs was used to perform PCA on the 203 unrelated individuals. We used rvtest^92^ to perform genotype-phenotype association analysis for 5082104 bi-allelic common SNPs with MAF > 5%. Sex, age, and the top 10 PCs were used as covariates for all association tests. The qqman^93^ and CMplot R packages^94^ were applied to generate Manhattan and quantile-quantile plots. We defined genome-wide significance for single-variant association tests at 5e^−8^, with suggestive significance at 1e^−6^.

### Gene expression analysis

RNA-seq raw sequencing reads were filtered by SOAPnuke^95^ to remove reads with sequencing adapters, low-quality base ratios (base quality < 5) >20%, and unknown base (‘N’ base) ratios >5%. Reads aligned to rRNA by Bowtie2 (v2.2.5)^96^ were removed. Clean reads were then mapped to the reference genome using HISAT2^97^. Bowtie2 (v2.2.5) was applied to align clean reads to the transcriptome. The gene expression level (FPKM) was determined by RSEM^98^. Genes with FPKM > 0.1 in at least one sample were retained. Differential expression analysis was performed using DESeq2 (v1.4.5)^99^. Differentially expressed genes were defined as those with a Benjamini-Hochberg adjusted *P* value < 0.05 and fold-change >2. GO enrichment analysis was performed using clusterProfiler^100^. GO Biological Process (BP) terms with an FDR adjusted *P* value threshold of 0.05 were considered as significant^101^.

Small RNA raw sequencing reads with low-quality tags (with more than four bases with quality <10 or more than six bases with quality <13), poly A tags, tags without a 3ʹ primer, or tags shorter than 18 nt were removed. After data filtering, the clean reads were mapped to the reference genome and other small RNA databases, including miRbase, siRNA, piRNA, and snoRNA using Bowtie2^96^. We performed cmsearch^102^ for Rfam mapping. Small RNA expression levels were calculated by counting absolute numbers of molecules using unique molecular identifiers (UMI, 8–10 nt).

### Construction of mRNA-miRNA and mRNA-lncRNA networks

To investigate post-transcriptional regulation, Spearman correlation coefficients of mRNA-miRNA (Supplementary Data 6) and mRNA-lncRNA were calculated (Supplementary Data 7). Correlation pairs with coefficients < -0.5 in mRNA-miRNA or <−0.6 in mRNA-lncRNA were retained. MultiMiR was used to confirm the top pairs of mRNA-miRNA by performing miRNA target prediction^103^. The mRNA-miRNA and mRNA-lncRNA networks were visualized using Cytoscape (Fig. 2d)^104^.

### Proteomics analysis

Serum samples were inactivated at 56 °C in a water bath for 30 min, followed by processing using a Cleanert PEP 96-well plate (Agela, China). According to the manufacturer’s instructions, high-abundance proteins under denaturing conditions were removed^105^. A Bradford Protein Assay Kit (Bio-Rad, USA) was used to determine the final protein concentration. Proteins were extracted by 8 M urea and subsequently reduced to a final concentration of 10 mM dithiothreitol in a 37 °C water bath for 30 min and alkylated to a final concentration of 55 mM iodoacetamide at room temperature for 30 min in a dark room. The extracted proteins were digested in trypsin (Promega, USA) with a 10 KD FASP filter (Sartorious, UK) at a protein-to-enzyme ratio of 50:1, then eluded with 70% acetonitrile (ACN) and dried in a freeze dryer.

Data independent acquisition (DIA) was performed using a Q Exactive HF mass spectrometer (Thermo Scientific, San Jose, USA) coupled with an UltiMate 3000 UHPLC liquid chromatograph (Thermo Scientific, San Jose, USA). Peptides (1 μg) mixed with iRT (Biognosys, Schlieren, Switzerland) were injected into the LC and enriched and desalted in the trap column. The peptides were then separated using a self-packed analytical column (150 μm internal diameter, 1.8 μm particle size, 35 cm column length) at a flow rate of 500 nL/min. The mobile phases consisted of (A) H_2_O/ACN (98/2,v/v) (0.1% formic acid); and (B) ACN/H_2_O (98/2,v/v) (0.1% formic acid) with 120 min elution gradient (min, %B): 0, 5; 5, 5; 45, 25; 50, 35; 52, 80; 55, 80; 55.5, 5; 65, 5. For HF settings, the ion source voltage was 1.9 kV and the MS1 range was 400–1 250 m/z at a resolution of 120 000 with a 50 ms max injection time (MIT). We divided 400–1 250 m/z equally into 45 continuous window MS2 scans at 30 000 resolution with the automatic MIT and automatic gain control (AGC) of 1E6. MS2 normalized collision energy was distributed to 22.5, 25, 27.5.

Raw data were analyzed using Spectronaut software (12.0.20491.14.21367) with the default settings against the self-built plasma spectral library to achieve deeper proteome quantification. The FDR cutoff for peptide and protein levels was set to 1%. The R package MSstats^106^ was used for log2 transformation, normalization, and P value calculation.

### Metabolomics analysis

Serum samples (100 μl) was transferred to 96-well plates and mixed with 10 μl of SPLASH LipidoMix^TM^ Internal Standard (Avanti Polar Lipids, USA) and 10 μl of home-made internal standard mixture containing D3-L-methionine (100 ppm, TRC, Canada), 13C9-phenylalanine (100 ppm, CIL, USA), D6-L-2-aminobutyric acid (100 ppm, TRC, Canada), D4-L-alanine (100 ppm, TRC, Canada), 13C4-L-threonine (100 ppm, CIL, USA), D3-L-aspartic acid (100 ppm, TRC, Canada), and 13C6-L-arginine (100 ppm, CIL, USA). Then, 300 μl of pre-chilled methanol/ACN extraction buffer (67/33, v/v) was added to the plasma sample, vortexed for 1 min, and incubated at −20 °C for 2 h. After centrifugation at 4000 rpm for 20 min, 300 μl of the supernatant was taken and freeze dried. The metabolites were dissolved in 150 μl of methanol/ACN buffer (50/50, v/v) and centrifuged at 4000 rpm for 30 min. The supernatants were then injected into the MS.

Metabolomics data acquisition was completed using the same spectrometry and LC and settings as used for lipidomics, except for the following parameters: the mobile phases of positive mode were (A) H_2_O (0.1% formic acid) and (B) methanol (0.1% formic acid). The mobile phases of negative mode were (A) H_2_O (10 mM NH_4_HCO_3_) and (B) methanol/H_2_O (95/5, v/v) (10 mM NH_4_HCO_3_). Both positive and negative models used the same gradient (min, %B): 0, 2; 1, 2; 9, 98; 12, 98; 12.1, 2; 15, 2. The temperature of the column was set at 45 °C. The MS1 range was set at 70–1 050 m/z. MS2 stepped normalized collision energy was distributed to 20, 40, 60.

Raw data were searched using Compound Discoverer v3.1 (Thermo Fisher Scientific, USA) with different libraries, including our self-built BGI library containing more than 3000 metabolites with corresponding detailed mass spectrum data. After quantification, subsequent processing steps were finished by metaX, the same as for lipidomics analysis.

### Lipidomics analysis

Serum samples (100 μl) were transferred to 96-well plates and mixed with 10 μl of SPLASH LipidoMix^TM^ Internal Standard (Avanti Polar Lipids, USA). We added 300 μl of pre-chilled isopropanol (IPA) to the plasma samples, which were then vortexed for 1 min and incubated at −20 °C overnight. The samples were then centrifuged at 4000 rpm for 20 min for protein precipitation. The supernatants were then used for MS analysis.

Lipidomics analysis was performed using a Q Exactive MS (Thermo Scientific, San Jose, USA) coupled with a Waters 2D UPLC (Waters, USA). The CSH C18 column (1.7 μm, 2.1 × 100 mm, Waters, USA) was used for separation with the following elution gradient (min, %B): (A) ACN/H_2_O (60/40, v/v) (10 mM NH_4_HCO_3_ and 0.1% formic acid) and (B) IPA/ACN (90/10, v/v) (10 mM NH_4_HCO_3_ and 0.1% formic acid): 0, 40; 2, 43; 2.1, 50; 7, 54; 7.1, 70; 13, 99; 13.1, 40; 15, 40. The column temperature was set at 55 °C, injection was set at 5 μL, and flow rate was set at 0.35 mL/min. For HF settings, the samples were scanned twice in both positive and negative modes. The positive and negative spray voltages were set to 3.80 kV and 3.20 kV, respectively. The MS1 range was 200–2 000 m/z at a resolution of 70,000 with 100 ms MIT and AGC of 3e6. The top3 precursors were set to trigger MS2 scans at a resolution of 17,500 with 50 ms MIT and AGC of 1E5. The MS2 stepped normalized collision energy was distributed to 15, 30, 45. The sheath gas flow rate was set at 40 and aux gas flow rate was set at 10.

Raw data were analyzed by LipidSearch v4.1 (Thermo Fisher Scientific, USA), including feature detection, identification, and alignment. The following settings were applied: tolerance of mass shift, 5 ppm; identification grade, A-D; filters, top rank; all isomer peak, FA priority, M-score, 5; c-score, 2.0. Export quantitative data from LipidSearch were analyzed using the R package metaX^107^, including normalization, correction of batch effects, and imputation of missing values.

### Differential expression of proteins, metabolites, and lipids

Expression data were first adjusted using the robust linear model (RLM). Following RLM, the residuals were analyzed using the two-sided Mann–Whitney rank test for each group pair and *P* values were adjusted using Benjamini-Hochberg. Differentially expressed proteins, metabolites, and lipids were defined based on an adjusted *P* value < 0.05 and absolute value of fold-change >1.5.

### Clustering

Clustering was performed using the R package ‘Mfuzz’. For mRNA from whole blood, differentially expressed genes were clustered. For proteins, metabolites, and lipids from serum, all three analytes were clustered together.

### Construction of gene regulatory network

ARACNe-AP^108^ was employed to construct gene regulatory networks (GRNs) for each group. The variability of gene expression traits was evaluated by median absolute deviation (MAD), and the top half of genes were recruited in the network. Mutual information^109^ was introduced to represent the strength of the regulatory relationship between TFs and target genes, and only significant pairs are retained (*P* < 1 × 10^-8^). We also executed 100 bootstraps and applied a data processing inequality tolerance filter^110^. The consensus network of each group was combined by statistically significant edges across all bootstrap networks (*P* < 0.05, Bonferroni-corrected), based on Poisson distribution. The degree was used to evaluate the centrality of genes in the network. To ensure the robustness of our remodeled GRN, we applied Chip-X Enrichment Analysis v3 (ChEA3)^111^ to identify TFs that target IFN and IFN receptors, with unrecognized ones eliminated.

### Quantification of cell fractions from bulk RNA-seq profiles

The estimation of abundances of immune cell types in blood tissue was performed using CIBERSORTx^112^ based on blood RNA-seq data.

### Protein interaction network construction and functional enrichment analysis

Interaction network construction and GO BP term enrichment of proteins were conducted using the STRING^113^ database with default parameters.

### Reporting summary

Further information on research design is available in the Nature Research Reporting Summary linked to this article.

## Supplementary information

Supplementary Information

Reporting Summary

Description of Additional Supplementary Files

Supplementary Data 1

Supplementary Data 2

Supplementary Data 3

Supplementary Data 4

Supplementary Data 5

Supplementary Data 6

Supplementary Data 7

Supplementary Data 8

Supplementary Data 9

## Data Availability

Source data are provided with this paper. The data that support the findings of this study have been deposited in European Bioinformatics Institute (EMBL-EBI). The transcriptome data have been deposited to EBI ENA with the study accession number ERP127339. The mass spectrometry proteomics data have been deposited to the EBI ProteomeXchange Consortium via the PRIDE partner repository with the dataset identifier PXD024674. The metabolites and lipids data have been deposited to EBI Metabolights with the data set identifier MTBLS2542. The genome association data have been deposited to EBI GWAS catalog with accession numbers (GCST90014052 accessible at [ftp.ebi.ac.uk:/pub/databases/gwas/summary_statistics/GCST90014001-GCST90015000/GCST90014052/GCST90014052_buildGRCh38.tsv.gz] and GCST90014053 accessible at [ftp.ebi.ac.uk:/pub/databases/gwas/summary_statistics/GCST90014001-GCST90015000/GCST90014053/GCST90014053_buildGRCh38.tsv.gz]). Custom scripts for data analysis in this study were present in [https://github.com/DongshengChen-TY/COVID19] (DOI: 10.5281/zenodo.4624526). Source data are provided with this paper.

## Source data

Source Data

## References

[1] WHO. WHO. Coronavirus disease (COVID-2019) situation report-160. 28 June 2020.) (2020).

[2] Hou, H. et al. Detection of IgM and IgG antibodies in patients with coronavirus disease 2019. *Clin. Transl. Immunol.* **9**, e01136 (2020). 10.1002/cti2.1136 PMC7202656 32382418

[3] Pan X (2020). Asymptomatic cases in a family cluster with SARS-CoV-2 infection. Lancet Infect. Dis..

[4] Chan JF (2020). A familial cluster of pneumonia associated with the 2019 novel coronavirus indicating person-to-person transmission: a study of a family cluster. Lancet..

[5] Bai Y (2020). Presumed asymptomatic carrier transmission of COVID-19. JAMA.

[6] Dong Y (2020). Epidemiology of COVID-19 among children in China. Pediatrics.

[7] Kimball A (2020). Asymptomatic and Presymptomatic SARS-CoV-2 Infections in Residents of a Long-Term Care Skilled Nursing Facility - King County, Washington, March 2020. MMWR Morb. Mortal Wkly. Rep..

[8] Wu Z, McGoogan JM (2020). Characteristics of and Important Lessons From the Coronavirus Disease 2019 (COVID-19) outbreak in China: summary of a Report of 72314 Cases From the Chinese Center for Disease Control and Prevention. JAMA.

[9] Zheng Z (2020). Risk factors of critical & mortal COVID-19 cases: a systematic literature review and meta-analysis. J. Infect.

[10] Gold MS (2020). COVID-19 and comorbidities: a systematic review and meta-analysis. Postgrad. Med..

[11] Wu C (2020). Risk factors associated with acute respiratory distress syndrome and death in patients with Coronavirus disease 2019 Pneumonia in Wuhan, China. JAMA Intern Med.

[12] Onder G, Rezza G, Brusaferro S (2020). Case-fatality rate and characteristics of patients dying in relation to COVID-19 in Italy. JAMA..

[13] Asfahan S. et al. Extrapolation of mortality in COVID-19: Exploring the role of age, sex, co-morbidities and health-care related occupation. *Monaldi Arch. Chest Dis.* **90**, (2020). 10.4081/monaldi.2020.1325 32447949

[14] Lu R (2020). Genomic characterisation and epidemiology of 2019 novel coronavirus: implications for virus origins and receptor binding. Lancet..

[15] Walls AC (2020). Structure, Function, and Antigenicity of the SARS-CoV-2 Spike Glycoprotein. Cell..

[16] Xiong Y (2020). Transcriptomic characteristics of bronchoalveolar lavage fluid and peripheral blood mononuclear cells in COVID-19 patients. Emerg. Microbes Infect..

[17] Wu D (2020). Plasma metabolomic and lipidomic alterations associated with COVID-19. Natl. Sci. Rev..

[18] Shen B (2020). Proteomic and metabolomic characterization of COVID-19 patient sera. Cell..

[19] Bojkova D (2020). Proteomics of SARS-CoV-2-infected host cells reveals therapy targets. Nature..

[20] Zhou F (2020). Clinical course and risk factors for mortality of adult inpatients with COVID-19 in Wuhan, China: a retrospective cohort study. Lancet..

[21] Guan, W. J. et al. Comorbidity and its impact on 1590 patients with COVID-19 in China: a nationwide analysis. *Eur. Respir. J.* **55**, 2000547 (2020). 10.1183/13993003.00547-2020 PMC7098485 32217650

[22] Zhang L (2018). The immunological regulation roles of porcine beta-1, 4 Galactosyltransferase V (B4GALT5) in PRRSV infection. Front Cell Infect. Microbiol..

[23] Silva-Filho JC, Melo CGF, Oliveira JL (2020). The influence of ABO blood groups on COVID-19 susceptibility and severity: a molecular hypothesis based on carbohydrate-carbohydrate interactions. Med Hypotheses..

[24] Ricciotti E, FitzGerald GA (2011). Prostaglandins and inflammation. Arterioscler Thromb. Vasc. Biol..

[25] Ellinghaus D (2020). Genomewide association study of severe Covid-19 with respiratory failure. N. Engl. J. Med..

[26] Liu S (2018). Genomic analyses from non-invasive prenatal testing reveal genetic associations, patterns of viral infections, and Chinese population history. Cell..

[27] Newman AM (2019). Determining cell type abundance and expression from bulk tissues with digital cytometry. Nat. Biotechnol..

[28] Litvak V (2012). FOXO3-IRF7 gene regulatory circuit limits inflammatory sequelae of antiviral responses. Nature..

[29] Sorgdrager FJH, Naude PJW, Kema IP, Nollen EA, Deyn PP (2019). Tryptophan metabolism in inflammaging: from biomarker to therapeutic target. Front Immunol..

[30] Moffett JR, Namboodiri MA (2003). Tryptophan and the immune response. Immunol. Cell Biol..

[31] Bronte V, Serafini P, Mazzoni A, Segal DM, Zanovello P (2003). L-arginine metabolism in myeloid cells controls T-lymphocyte functions. Trends Immunol..

[32] Xu K, Nagy PD (2015). RNA virus replication depends on enrichment of phosphatidylethanolamine at replication sites in subcellular membranes. Proc. Natl. Acad. Sci. USA.

[33] Marichal-Cancino BA, Fajardo-Valdez A, Ruiz-Contreras AE, Mendez-Diaz M, Prospero-Garcia O (2017). Advances in the physiology of GPR55 in the central nervous system. Curr. Neuropharmacol..

[34] Avota E, Schneider-Schaulies S (2014). The role of sphingomyelin breakdown in measles virus immunmodulation. Cell Physiol. Biochem..

[35] Avota E, Gulbins E, Schneider-Schaulies S (2011). DC-SIGN mediated sphingomyelinase-activation and ceramide generation is essential for enhancement of viral uptake in dendritic cells. PLoS Pathog..

[36] Drobnik W (2003). Plasma ceramide and lysophosphatidylcholine inversely correlate with mortality in sepsis patients. J. Lipid Res.

[37] Yan JJ (2004). Therapeutic effects of lysophosphatidylcholine in experimental sepsis. Nat. Med..

[38] Jin Y, Knudsen E, Wang L, Maghazachi AA (2003). Lysophosphatidic acid induces human natural killer cell chemotaxis and intracellular calcium mobilization. Eur. J. Immunol..

[39] Galani IE, Andreakos E (2015). Neutrophils in viral infections: current concepts and caveats. J. Leukoc. Biol..

[40] Papayannopoulos V (2018). Neutrophil extracellular traps in immunity and disease. Nat. Rev. Immunol..

[41] Middleton EA (2020). Neutrophil Extracellular Traps (NETs) contribute to immunothrombosis in COVID-19 acute respiratory distress syndrome. Blood..

[42] Long QX (2020). Clinical and immunological assessment of asymptomatic SARS-CoV-2 infections. Nat. Med..

[43] Mino T, Takeuchi O (2018). Post-transcriptional regulation of immune responses by RNA binding proteins. Proc. Jpn Acad. Ser. B Phys. Biol. Sci..

[44] Tanaka T, Narazaki M, Kishimoto T (2014). IL-6 in inflammation, immunity, and disease. Cold Spring Harb. Perspect. Biol..

[45] Carpenter S, Ricci EP, Mercier BC, Moore MJ, Fitzgerald KA (2014). Post-transcriptional regulation of gene expression in innate immunity. Nat. Rev. Immunol..

[46] Xu J (2018). Circulating plasma extracellular vesicles from septic mice induce inflammation via MicroRNA- and TLR7-dependent mechanisms. J. Immunol..

[47] Vaher H (2019). miR-10a-5p is increased in atopic dermatitis and has capacity to inhibit keratinocyte proliferation. Allergy..

[48] Yang Q (2019). Downregulation of microRNA-23b-3p alleviates IL-1beta-induced injury in chondrogenic CHON-001 cells. Drug Des. Devel Ther..

[49] Grifoni A (2020). Targets of T cell responses to SARS-CoV-2 Coronavirus in humans with COVID-19 disease and unexposed individuals. Cell.

[50] Munn DH (2005). GCN2 kinase in T cells mediates proliferative arrest and anergy induction in response to indoleamine 2,3-dioxygenase. Immunity.

[51] Werner A (2017). Reconstitution of T cell proliferation under arginine limitation: activated human T cells take up citrulline via L-type amino acid transporter 1 and use it to regenerate arginine after induction of argininosuccinate synthase expression. Front Immunol..

[52] Bost P (2020). Host-viral infection maps reveal signatures of severe COVID-19 patients. Cell..

[53] Blanco-Melo D (2020). Imbalanced host response to SARS-CoV-2 drives development of COVID-19. Cell..

[54] Broggi A (2020). Type III interferons disrupt the lung epithelial barrier upon viral recognition. Science.

[55] Tezcan G (2019). MicroRNA post-transcriptional regulation of the NLRP3 inflammasome in immunopathologies. Front Pharm..

[56] Csoka B (2018). Adenosine receptors differentially regulate type 2 cytokine production by IL-33-activated bone marrow cells, ILC2s, and macrophages. FASEB J..

[57] Srivastava, R., Daulatabad, S. V., Srivastava, M., Janga, S. C. Role of SARS-CoV-2 in altering the RNA-binding protein and miRNA-directed post-transcriptional regulatory networks in humans. *Int. J. Mol. Sci*. **21**, 7090 (2020). 10.3390/ijms21197090 PMC7582926 32993015

[58] Cathcart AL, Rozovics JM, Semler BL (2013). Cellular mRNA decay protein AUF1 negatively regulates enterovirus and human rhinovirus infections. J. Virol..

[59] Sadri N, Schneider RJ (2009). Auf1/Hnrnpd-deficient mice develop pruritic inflammatory skin disease. J. Invest Dermatol..

[60] Taylor GA (1996). A pathogenetic role for TNF alpha in the syndrome of cachexia, arthritis, and autoimmunity resulting from tristetraprolin (TTP) deficiency. Immunity..

[61] Garg AV (2015). MCPIP1 endoribonuclease activity negatively regulates interleukin-17-mediated signaling and inflammation. Immunity..

[62] Omiya S (2020). Cytokine mRNA degradation in cardiomyocytes restrains sterile inflammation in pressure-overloaded hearts. Circulation..

[63] Tahamtan A, Teymoori-Rad M, Nakstad B, Salimi V (2018). Anti-inflammatory MicroRNAs and their potential for inflammatory diseases treatment. Front Immunol..

[64] Tate MD, Brooks AG, Reading PC (2008). The role of neutrophils in the upper and lower respiratory tract during influenza virus infection of mice. Respir. Res..

[65] Fujisawa H (2008). Neutrophils play an essential role in cooperation with antibody in both protection against and recovery from pulmonary infection with influenza virus in mice. J. Virol..

[66] Oved JH (2021). Neutrophils promote clearance of nuclear debris following acid-induced lung injury. Blood..

[67] Tsai YF (2018). Garcinia Multiflora Inhibits FPR1-Mediated Neutrophil Activation and Protects Against Acute Lung Injury. Cell Physiol. Biochem..

[68] Wilk AJ (2020). A single-cell atlas of the peripheral immune response in patients with severe COVID-19. Nat. Med..

[69] Narasaraju T (2011). Excessive neutrophils and neutrophil extracellular traps contribute to acute lung injury of influenza pneumonitis. Am. J. Pathol..

[70] Sollberger, G. et al. Gasdermin D plays a vital role in the generation of neutrophil extracellular traps. *Sci. Immunol*. **3**, eear6689 (2018). 10.1126/sciimmunol.aar6689 30143555

[71] Thiam HR (2020). NETosis proceeds by cytoskeleton and endomembrane disassembly and PAD4-mediated chromatin decondensation and nuclear envelope rupture. Proc. Natl. Acad. Sci. USA.

[72] Polverino E, Rosales-Mayor E, Dale GE, Dembowsky K, Torres A (2017). The role of neutrophil elastase inhibitors in lung diseases. Chest..

[73] Diao B (2020). Reduction and functional exhaustion of T cells in patients with Coronavirus disease 2019 (COVID-19). Front Immunol..

[74] Fathi N., Rezaei N. Lymphopenia in COVID-19: therapeutic opportunities. *Cell Biol. Int.* **44**, 1792-1797 (2020). 10.1002/cbin.11403 PMC7283672 32458561

[75] Mullard A (2018). IDO takes a blow. Nat. Rev. Drug Disco..

[76] Zhai L (2015). Molecular pathways: targeting IDO1 and other tryptophan dioxygenases for cancer immunotherapy. Clin. Cancer Res..

[77] Cronin SJF (2018). The metabolite BH4 controls T cell proliferation in autoimmunity and cancer. Nature..

[78] Darcy CJ (2014). Neutrophils with myeloid derived suppressor function deplete arginine and constrain T cell function in septic shock patients. Crit. Care..

[79] Arunachalam PS (2020). Systems biological assessment of immunity to mild versus severe COVID-19 infection in humans. Science..

[80] Shibabaw T, Molla MD, Teferi B, Ayelign B (2020). Role of IFN and Complements System: Innate Immunity in SARS-CoV-2. J. Inflamm. Res..

[81] Opitz CA (2020). The therapeutic potential of targeting tryptophan catabolism in cancer. Br. J. Cancer..

[82] Wang LT (2017). Intestine-specific homeobox gene ISX integrates IL6 signaling, tryptophan catabolism, and immune suppression. Cancer Res..

[83] Kim KD (2007). Adaptive immune cells temper initial innate responses. Nat. Med.

[84] Ricciuti B (2019). Targeting indoleamine-2,3-dioxygenase in cancer: Scientific rationale and clinical evidence. Pharm. Ther..

[85] Gunther J, Dabritz J, Wirthgen E (2019). Limitations and off-target effects of tryptophan-related IDO inhibitors in cancer treatment. Front Immunol..

[86] Crosignani S (2017). Discovery of a novel and selective indoleamine 2,3-dioxygenase (IDO-1) inhibitor 3-(5-Fluoro-1H-indol-3-yl)pyrrolidine-2,5-dione (EOS200271/PF-06840003) and its characterization as a potential clinical candidate. J. Med Chem..

[87] Waldman AD, Fritz JM, Lenardo MJ (2020). A guide to cancer immunotherapy: from T cell basic science to clinical practice. Nat. Rev. Immunol.

[88] Freed, D., Aldana, R., Weber, J. A., Edwards, J. S. The Sentieon Genomics Tools - A fast and accurate solution to variant calling from next-generation sequence data. *bioRxiv.*10.1101/115717 (2017).

[89] Li H, Durbin R (2009). Fast and accurate short read alignment with Burrows-Wheeler transform. Bioinformatics..

[90] Van der Auwera GA (2013). From FastQ data to high confidence variant calls: the Genome Analysis Toolkit best practices pipeline. Curr. Protoc. Bioinforma..

[91] Chang CC (2015). Second-generation PLINK: rising to the challenge of larger and richer datasets. Gigascience..

[92] Zhan X, Hu Y, Li B, Abecasis GR, Liu DJ (2016). RVTESTS: an efficient and comprehensive tool for rare variant association analysis using sequence data. Bioinformatics..

[93] Turner, S. D. qqman: an R package for visualizing GWAS results using Q-Q and manhattan plots. *Biorxiv.* (2014).

[94] Yin, L. CMplot: https://github.com/YinLiLin/R-CMplot (2020).

[95] Li R, Li Y, Kristiansen K, Wang J (2008). SOAP: short oligonucleotide alignment program. Bioinformatics..

[96] Langmead B, Salzberg SL (2012). Fast gapped-read alignment with Bowtie 2. Nat. Methods..

[97] Kim D, Langmead B, Salzberg SL (2015). HISAT: a fast spliced aligner with low memory requirements. Nat. Methods..

[98] Li B, Dewey CN (2011). RSEM: accurate transcript quantification from RNA-Seq data with or without a reference genome. BMC Bioinforma..

[99] Love MI, Huber W, Anders S (2014). Moderated estimation of fold change and dispersion for RNA-seq data with DESeq2. Genome Biol..

[100] Yu G, Wang LG, Han Y, He QY (2012). clusterProfiler: an R package for comparing biological themes among gene clusters. OMICS..

[101] Abdi H. The Bonferonni and Šidák corrections for multiple comparisons. Encycl Meas Stat. **3**, (2007).

[102] Nawrocki EP, Eddy SR (2013). Infernal 1.1: 100-fold faster RNA homology searches. Bioinformatics..

[103] Ru Y (2014). The multiMiR R package and database: integration of microRNA-target interactions along with their disease and drug associations. Nucleic Acids Res..

[104] Shannon P (2003). Cytoscape: a software environment for integrated models of biomolecular interaction networks. Genome Res..

[105] Lin Z (2020). Evaluation and minimization of nonspecific tryptic cleavages in proteomic sample preparation. Rapid Commun. Mass Spectrom..

[106] Choi M (2014). MSstats: an R package for statistical analysis of quantitative mass spectrometry-based proteomic experiments. Bioinformatics..

[107] Wen B, Mei Z, Zeng C, Liu S (2017). metaX: a flexible and comprehensive software for processing metabolomics data. BMC Bioinforma..

[108] Lachmann A, Giorgi FM, Lopez G, Califano A (2016). ARACNe-AP: gene network reverse engineering through adaptive partitioning inference of mutual information. Bioinformatics..

[109] Steuer R, Kurths J, Daub CO, Weise J, Selbig J (2002). The mutual information: detecting and evaluating dependencies between variables. Bioinformatics..

[110] Margolin AA (2006). ARACNE: an algorithm for the reconstruction of gene regulatory networks in a mammalian cellular context. BMC Bioinforma..

[111] Keenan AB (2019). ChEA3: transcription factor enrichment analysis by orthogonal omics integration. Nucleic Acids Res..

[112] Chen B, Khodadoust MS, Liu CL, Newman AM, Alizadeh AA (2018). Profiling Tumor Infiltrating Immune Cells with CIBERSORT. Methods Mol. Biol..

[113] Szklarczyk D (2019). STRING v11: protein-protein association networks with increased coverage, supporting functional discovery in genome-wide experimental datasets. Nucleic Acids Res.

