# Supplementary material for: The trans-omics landscape of COVID-19
Source: Nat Commun. 2021 Jul 27;12:4543. doi: 10.1038/s41467-021-24482-1 (PMC8316550; doi:10.1038/s41467-021-24482-1)
Supplement: Supplementary file 1 — Supplementary Information [file 41467_2021_24482_MOESM1_ESM.pdf]

Supplementary Information for

The Trans-omics Landscape of COVID-19

Peng Wu[1,2†], Dongsheng Chen[3†], Wencheng Ding[1,2†], Ping Wu[1,†], Hongyan Hou[5†], Yong Bai[3†], Yuwen Zhou[3,4†], Kezhen Li[1,2†], Shunian Xiang[3], Panhong Liu[3], Jia Ju[3,4], Ensong Guo[1,2], Jia Liu[1,2], Bin Yang[1,2], Junpeng Fan[1], Liang He[1], Ziyong Sun[5], Ling Feng[6], Jian Wang[7], Tangchun Wu[8], Hao Wang[8], Jin Cheng[9], Hui Xing[10], Yifan Meng[11], Yongsheng Li[12], Yuanliang Zhang[3], Hongbo Luo[3,13], Gang Xie[3,4], Xianmei Lan[3], Ye Tao[3], Jiafeng Li[3,4], Hao Yuan[3,4], Kang Huang[3], Wan Sun[3], Xiaobo Qian[3,4] Zhichao Li[3,4], Mingxi Huang[3,4], Peiwen Ding[3,4], Haoyu Wang[3,4], Jiaying Qiu[3,4], Feiyue Wang[3,4], Shiyou Wang[3,4], Jiacheng Zhu[3,4], Xiangning Ding[3,4], Chaochao Chai[3], Langchao Liang[3], Xiaoling Wang[3,4], Lihua Luo[3,4], Yuzhe Sun[3], Ying Yang[3], Zhenkun Zhuang[3,14], Tao Li[3], Lei Tian[3], Shaoqiao Zhang[15], Linnan Zhu[3], Ashley Chang[3], Lei Chen[16], Songchen Yang[3], Chengcheng Sun[3], Yanan Zhang[17], Yi Jia[3], Ya Gao[3], Fang Chen[3], Yan Ren[3], Xun Xu[3,18], Siqi Liu[3], Jian Wang[3,19], Huanming Yang[3,19], Lin Wang[7]*, Chaoyang Sun[1,2]*, Ding Ma[1,2]*, Xin Jin[3,20,21]*, Gang Chen[1,2]*

1. Cancer Biology Research Center (Key Laboratory of the Ministry of Education), Tongji Medical College, Tongji Hospital, Huazhong University of Science and Technology, Wuhan 430030, China
2. Department of Gynecologic Oncology, Tongji Hospital, Tongji Medical College, Huazhong University of Science and Technology, Wuhan 430030, China
3. BGI-Shenzhen, Shenzhen 518083, China
4. College of Life Sciences, University of Chinese Academy of Sciences, Beijing 100049, China
5. Department of Laboratory Medicine, Tongji Hospital, Tongji Medical College, Huazhong University of Science and Technology, Wuhan 430030, China
6. Department of Gynecology and Obstetrics, Tongji Hospital, Tongji Medical College, Huazhong University of Science & Technology, Wuhan 430030, China
7. Department of Clinical Laboratory, Union Hospital, Tongji Medical College, Huazhong University of Science and Technology, Wuhan 430022, China
8. Department of Occupational and Environmental Health, Key Laboratory of Environment and Health, Ministry of Education and State Key Laboratory of Environmental Health (Incubating), School of Public Health, Tongji Medical College, Huazhong University of Science and Technology, Wuhan 430030, China
9. Department of Research, Xiangyang Central Hospital, Hubei University of Arts and Science, Xiangyang, Hubei 441021, China
10. Department of Obstetrics and Gynecology, Xiangyang Central Hospital, Hubei University of Arts and Science, Xiangyang, Hubei 441021, China
11. Department of Gynecologic Oncology, State Key Laboratory of Oncology in South China, Collaborative Innovation Center for Cancer Medicine, Sun Yat-Sen University Cancer Center, Guangzhou 510060, China
12. Key Laboratory of Tropical Translational Medicine of Ministry of Education, Hainan Medical University, Haikou 571199, China
13. BGI-Guizhou, BGI-Shenzhen, Guiyang 550014, China
14. School of Biology and Biological Engineering, South China University of Technology, Guangzhou 510006, China
15. BGI-Hubei, BGI-Shenzhen, Wuhan 430074, China
16. College of Veterinary Medicine, Yangzhou University, Yangzhou 225009, China

17. Tsinghua-Berkeley Shenzhen Institute, Tsinghua University, Shenzhen 518055, China
18. Guangdong Provincial Key Laboratory of Genome Read and Write, BGI-Shenzhen, Shenzhen 518120, China
19. James D. Watson Institute of Genome Science, Hangzhou 310008, China
20. School of Medicine, South China University of Technology, Guangzhou, Guangdong 510006, China
21. Guangdong Provincial Key Laboratory of Human Disease Genomics, Shenzhen Key Laboratory of Genomics, BGI-Shenzhen, Shenzhen 518083, China

† These authors contributed equally to this work.
* Correspondence should be addressed to:
Gang Chen (tjchengang@hust.edu.cn)
Xin Jin (jinxin@genomics.cn)
Ding Ma (Dingma424@126.com)
Chaoyang Sun (suncydoctor@gmail.com)
Lin Wang (lin_wang@hust.edu.cn)

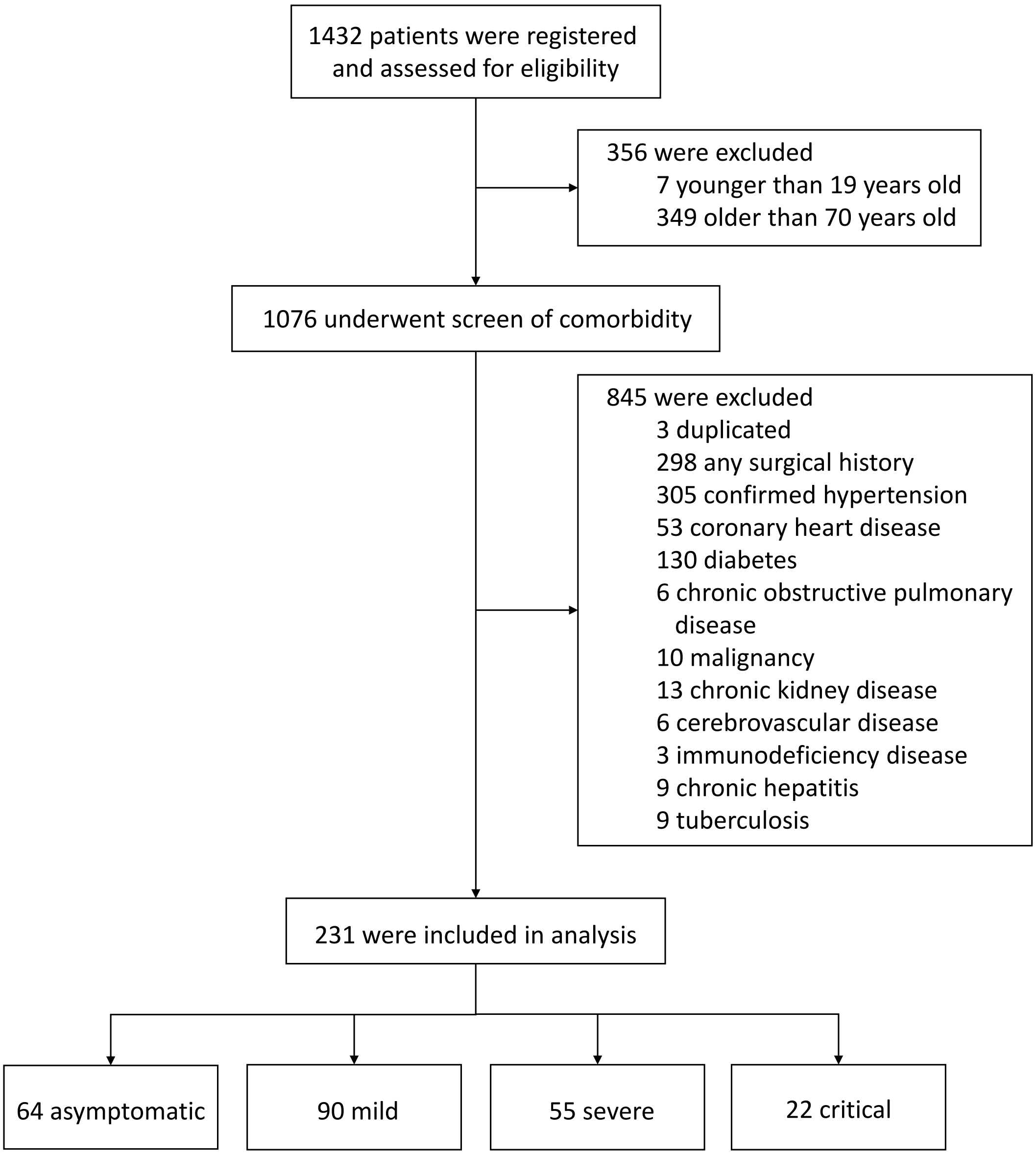

Supplementary Fig. 1 Study design for multi-omics analysis

All COVID-19 patients (n = 1432) enrolled in this study were screened to exclude those with selected complications, operation history, or aged younger than 19 or older than 70. After filtering, 231 patients were enrolled and grouped as asymptomatic (n = 64), mild (n = 90), severe (n = 55), or critical (n = 22). Sample DNA, RNA, and total serum proteins and lipids were isolated, followed by WGS, RNA-seq, and LC-MS analyses, respectively, and multi-omics integration analysis.

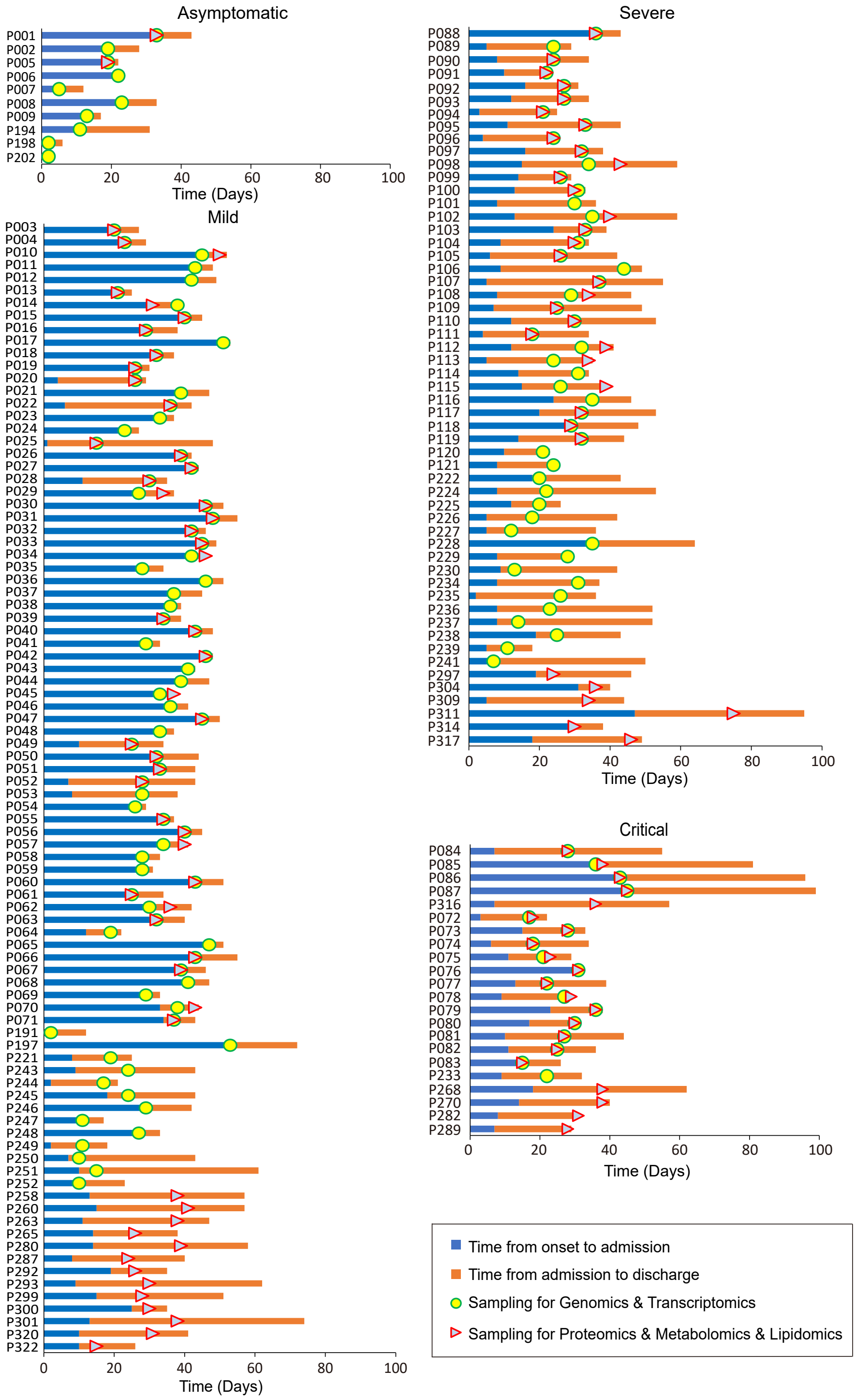

Supplementary Fig. 2 Clinical characteristics of enrolled patients

Summary of clinical characteristics of hospitalized COVID-19 patients, including asymptomatic, mild, severe, and critical. For asymptomatic patients, only 10 were hospitalized, with others diagnosed at the clinic and isolated at home. Patient IDs were labeled along y-axis.

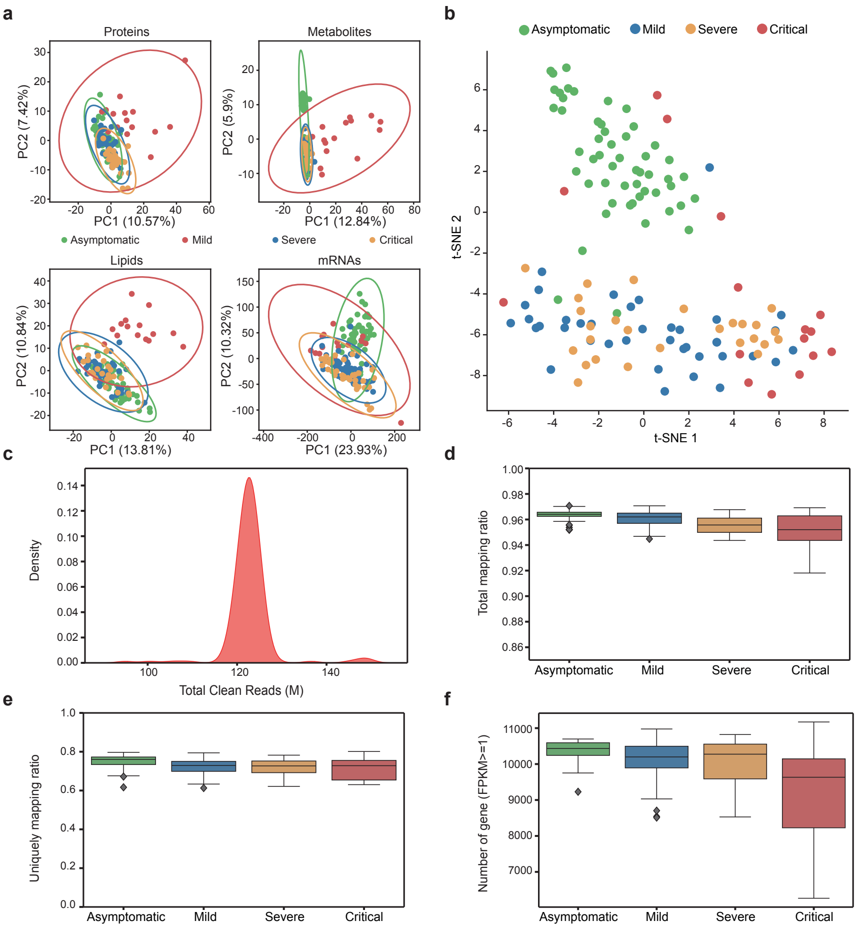

Supplementary Fig. 3 Quality control for data generation

a, Principal component analysis of proteomics, metabolomics, lipidomics, and transcriptomics. The transcriptomics analysis included asymptomatic (n = 64), mild (n = 64), severe (n = 34), and critical (n = 16) COVID-19 patients. The proteins, metabolites, and lipids analysis included asymptomatic (n = 53), mild (n = 54), severe (n = 33), and critical (n = 21) COVID-19 patients. **b,** 2D visualization of all multi-omics analytes using t-distributed stochastic neighbor embedding (tSNE) algorithm. Each dot represents a single sample. **c,** Density plot of total clean reads for all samples. **d,** Boxplot of total mapping ratio across four disease groups. **e,** Unique mapping ratio. **f,** Boxplot of detected gene numbers (FPKM ≥ 1) across four disease groups. The bold lines, upper boundaries and lower boundaries of notches represent the medians, 75th percentiles and 25th percentiles, respectively. Whiskers extend 1.5 times interquartile range (IQR). Data beyond the whiskers are considered as outliers and plotted as individual points.

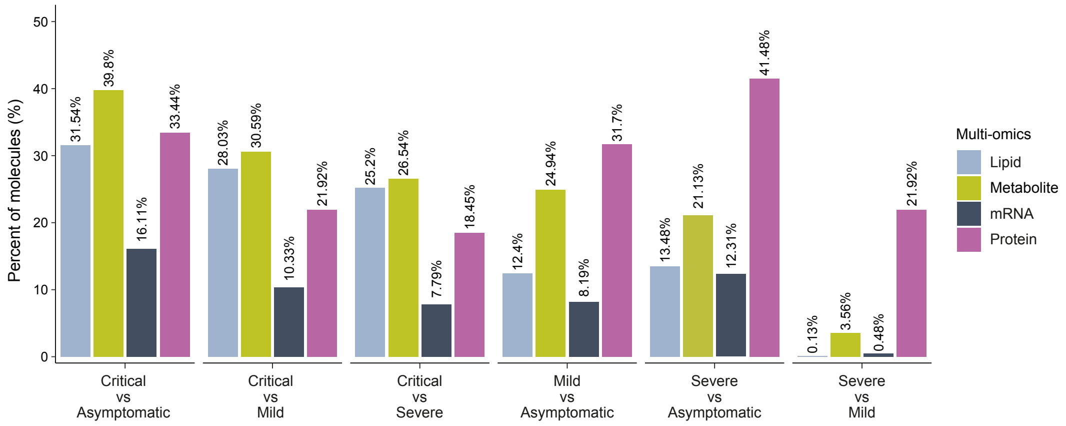

Supplementary Fig. 4 Bar graph representing proportion of analytes across molecule types showing significant differential expression between any two disease statuses

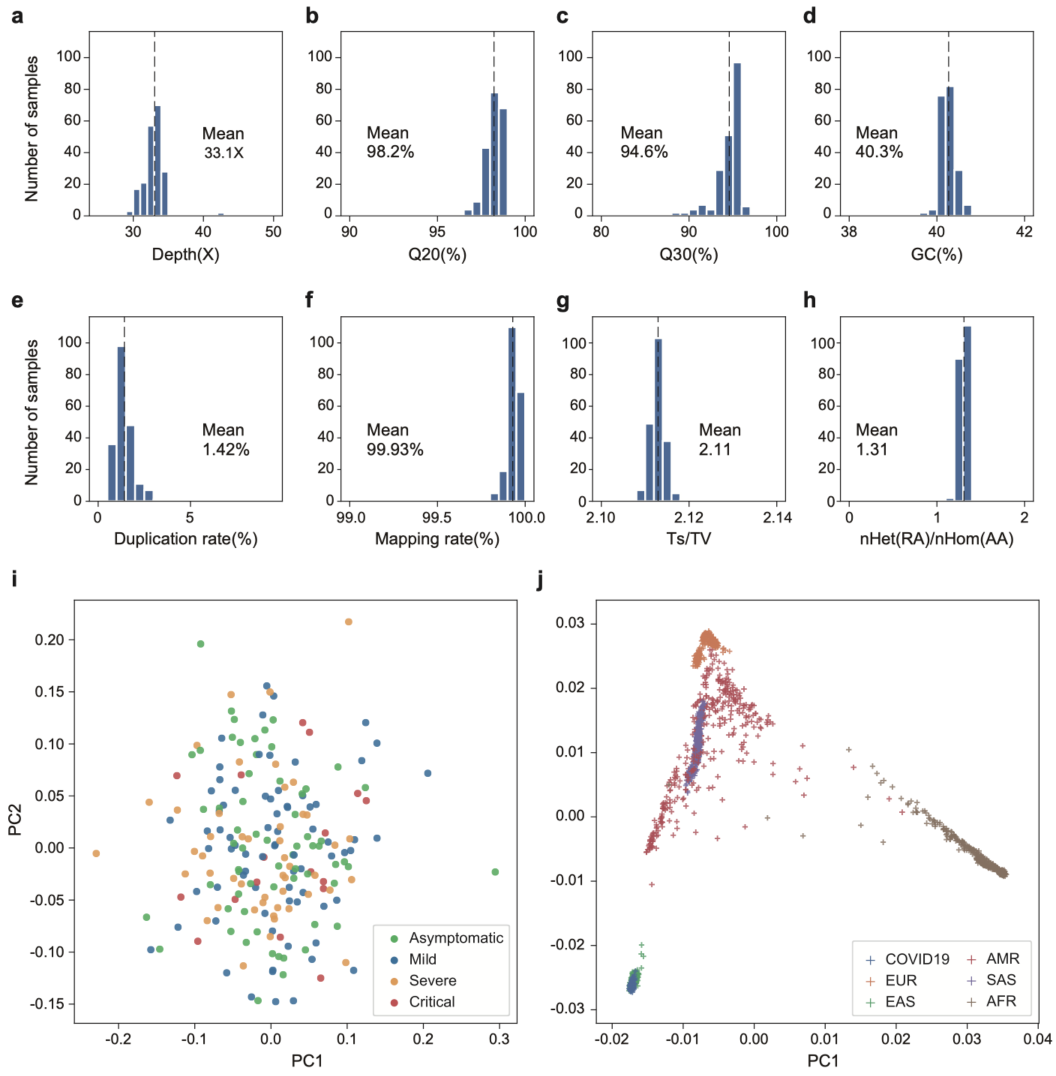

Supplementary Fig. 5 Quality control of 203 sequenced genomes and principal component analysis (PCA)

a-f, Quality control matrix for genome sequencing and mapping. **g-h,** Ratio of transitions to transversions (Ts/Tv) and ratio of heterozygous sites to non-reference homozygous sites for each patient. **i,** PCA based on genotype of 203 unrelated individuals, showing PC1 vs. PC2. **j,** PCA together with 2504 individuals from 1000 Genome Project, with all 203 COVID-19 patients grouped within the East Asian population.

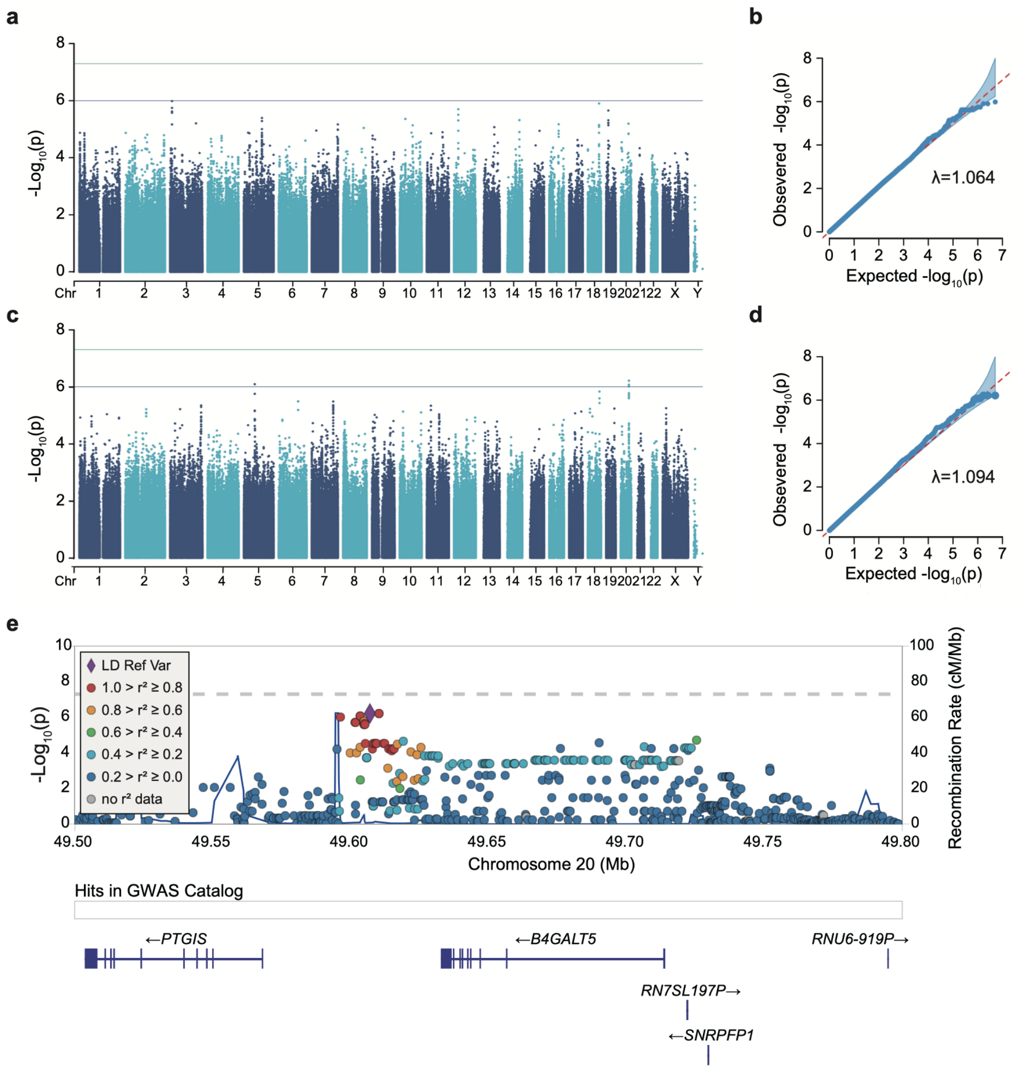

Supplementary Fig. 6 Association study results

Single-variant association test for common variants (MAF > 0.05). **a,** Manhattan plot and **b,** QQ-plot for severe and critical groups (n = 65) versus asymptomatic and mild groups (n = 138). P values were calculated using two-sided score test. **c,** Manhattan plot and **d,** QQ-plot for asymptomatic group (n = 63) versus symptomatic groups (n = 140). P values were calculated using two-sided score test. **e,** Locus zoom for genomic region flanking suggestive signal associated with absence of symptoms: showing chr20:49.5M-49.8M.

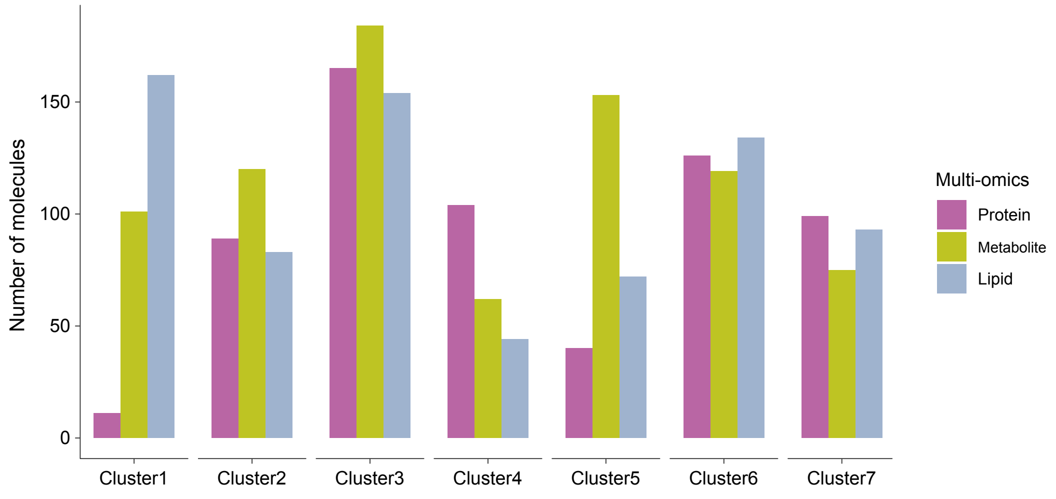

Supplementary Fig. 7 Cluster composition of proteins, metabolites, and lipids
