# Supplementary material for: The trans-omics landscape of COVID-19
Source: Nat Commun. 2021 Jul 27;12:4543. doi: 10.1038/s41467-021-24482-1 (PMC8316550; doi:10.1038/s41467-021-24482-1)
Supplement: Supplementary file 2 — Reporting Summary [file 41467_2021_24482_MOESM2_ESM.pdf]

nature research

Corresponding author(s): Gang Chen (tjchengang@hust.edu.cn)
Xin Jin (jinxin@genomics.cn)
Ding Ma (Dingma424@126.com)
Chaoyang Sun (suncydoctor@gmail.com)
Lin Wang (lin_wang@hust.edu.cn)

Last updated by author(s): Apr 26, 2021

Reporting Summary

Nature Research wishes to improve the reproducibility of the work that we publish. This form provides structure for consistency and transparency in reporting. For further information on Nature Research policies, see our Editorial Policies and the Editorial Policy Checklist.

Statistics

For all statistical analyses, confirm that the following items are present in the figure legend, table legend, main text, or Methods section.

n/a	Confirmed	
☐	☒	The exact sample size (*n*) for each experimental group/condition, given as a discrete number and unit of measurement
☐	☒	A statement on whether measurements were taken from distinct samples or whether the same sample was measured repeatedly
☐	☒	The statistical test(s) used AND whether they are one- or two-sided<br>*Only common tests should be described solely by name; describe more complex techniques in the Methods section.*
☐	☒	A description of all covariates tested
☐	☒	A description of any assumptions or corrections, such as tests of normality and adjustment for multiple comparisons
☐	☒	A full description of the statistical parameters including central tendency (e.g. means) or other basic estimates (e.g. regression coefficient) AND variation (e.g. standard deviation) or associated estimates of uncertainty (e.g. confidence intervals)
☐	☒	For null hypothesis testing, the test statistic (e.g. *F*, *t*, *r*) with confidence intervals, effect sizes, degrees of freedom and *P* value noted<br>*Give P values as exact values whenever suitable.*
☒	☐	For Bayesian analysis, information on the choice of priors and Markov chain Monte Carlo settings
☒	☐	For hierarchical and complex designs, identification of the appropriate level for tests and full reporting of outcomes
☐	☒	Estimates of effect sizes (e.g. Cohen's *d*, Pearson's *r*), indicating how they were calculated

Our web collection on statistics for biologists contains articles on many of the points above.

Software and code

Policy information about availability of computer code

Data collection	No software was used.
Data analysis	Whole-genome sequencing data analysis and joint variant calling<br>Whole-genome sequencing data were processed using Sentieon Genomics (v: sentieon-genomics-201911)85. The pipeline was built according to best practice workflows for germline short variant discovery described in https://gatk.broadinstitute.org/. Sequencing reads were mapped to the hg38 reference genome using the BWA algorithm86. After duplicate marking, Indel realignment, and base quality score recalibration (BQSR), per-sample variants were called using the Haplotyper algorithm in GVCF mode. The GVCFtyper algorithm was then used to perform joint-calling and generate cohort VCF. Variant quality score recalibration was performed using the Genome Analysis Toolkit (GATK v4.1.2)87. The truth-sensitivity-filter-level was set to 99.0 for both SNPs and Indels. Finally, variants with a PASS flag and quality score ≥ 100 were selected for further analysis.<br><br>Genotype-phenotype association analysis<br>Principal component analysis (PCA) was performed using PLINK (v1.9)88. Bi-allelic SNPs were selected based on the following criteria: MAF ≥ 5%; genotyping rate ≥ 90%; LD prune (window = 50, step = 5 and r2 ≥ 0.5). A subset of 605867 SNPs was used to perform PCA on the 203 unrelated individuals. We used rvtest89 to perform genotype-phenotype association analysis for 5082104 bi-allelic common SNPs with MAF > 5%. Sex, age, and the top 10 PCs were used as covariates for all association tests. The qqman90 and CMplot R packages91 were applied to generate Manhattan and quantile-quantile plots. We defined genome-wide significance for single-variant association tests at 5e-8, with suggestive significance at 1e-6.<br><br>Gene expression analysis<br>RNA-seq raw sequencing reads were filtered by SOAPnuke92 to remove reads with sequencing adapters, low-quality base ratios (base quality < 5) > 20%, and unknown base ('N' base) ratios > 5%. Reads aligned to rRNA by Bowtie2 (v2.2.5)93 were removed. Clean reads were then

mapped to the reference genome using HISAT294. Bowtie2 (v2.2.5) was applied to align clean reads to the transcriptome. The gene expression level (FPKM) was determined by RSEM95. Genes with FPKM > 0.1 in at least one sample were retained. Differential expression analysis was performed using DESeq2 (v1.4.5) with sex and age as confounders96. Differentially expressed genes were defined as those with a Benjamini-Hochberg adjusted P value < 0.05 and fold-change > 2. GO enrichment analysis was performed using clusterProfiler97. GO Biological Process (BP) terms with an FDR adjusted P value threshold of 0.05 were considered as significant98.

Small RNA raw sequencing reads with low-quality tags (with more than four bases with quality < 10 or more than six bases with quality < 13), poly A tags, tags without a 3' primer, or tags shorter than 18 nt were removed. After data filtering, the clean reads were mapped to the reference genome and other small RNA databases, including miRbase, siRNA, piRNA, and snoRNA using Bowtie293. We performed cmsearch99 for Rfam mapping. Small RNA expression levels were calculated by counting absolute numbers of molecules using unique molecular identifiers (UMI, 8–10 nt).

Construction of mRNA-miRNA and mRNA-lncRNA networks
To investigate post-transcriptional regulation, Spearman correlation coefficients of mRNA-miRNA (Supplementary Data 6) and mRNA-lncRNA were calculated (Supplementary Data 7). Correlation pairs with coefficients < -0.5 in mRNA-miRNA or < -0.6 in mRNA-lncRNA were retained. MultiMiR was used to confirm the top pairs of mRNA-miRNA by performing miRNA target prediction100. The mRNA-miRNA and mRNA-lncRNA networks were visualized using Cytoscape (Fig. 2d)101.

Proteomics analysis
Serum samples were inactivated at 56 °C in a water bath for 30 min, followed by processing using a Cleanert PEP 96-well plate (Agela, China). According to the manufacturer's instructions, high-abundance proteins under denaturing conditions were removed102. A Bradford Protein Assay Kit (Bio-Rad, USA) was used to determine the final protein concentration. Proteins were extracted by 8 M urea and subsequently reduced to a final concentration of 10 mM dithiothreitol in a 37 °C water bath for 30 min and alkylated to a final concentration of 55 mM iodoacetamide at room temperature for 30 min in a dark room. The extracted proteins were digested in trypsin (Promega, USA) with a 10 KD FASP filter (Sartorious, UK) at a protein-to-enzyme ratio of 50:1, then eluded with 70% acetonitrile (ACN) and dried in a freeze dryer.

Data independent acquisition (DIA) was performed using a Q Exactive HF mass spectrometer (Thermo Scientific, San Jose, USA) coupled with an UltiMate 3000 UHPLC liquid chromatograph (Thermo Scientific, San Jose, USA). Peptides (1 μg) mixed with iRT (Biognosys, Schlieren, Switzerland) were injected into the LC and enriched and desalted in the trap column. The peptides were then separated using a self-packed analytical column (150 μm internal diameter, 1.8 μm particle size, 35 cm column length) at a flow rate of 500 nL/min. The mobile phases consisted of (A) H2O/ACN (98/2,v/v) (0.1% formic acid); and (B) ACN/H2O (98/2,v/v) (0.1% formic acid) with 120 min elution gradient (min, % B): 0, 5; 5, 5; 45, 25; 50, 35; 52, 80; 55, 80; 55.5, 5; 65, 5. For HF settings, the ion source voltage was 1.9 kV and the MS1 range was 400–1250 m/z at a resolution of 120000 with a 50 ms max injection time (MIT). We divided 400–1250 m/z equally into 45 continuous window MS2 scans at 30000 resolution with the automatic MIT and automatic gain control (AGC) of 1E6. MS2 normalized collision energy was distributed to 22.5, 25, 27.5.

Raw data were analyzed using Spectronaut software (12.0.20491.14.21367) with the default settings against the self-built plasma spectral library to achieve deeper proteome quantification. The FDR cutoff for peptide and protein levels was set to 1%. The R package MSstats103 was used for log2 transformation, normalization, and P value calculation.

Metabolomics analysis
Serum samples (100 μl) was transferred to 96-well plates and mixed with 10 μl of SPLASH LipidoMixTM Internal Standard (Avanti Polar Lipids, USA) and 10 μl of home-made internal standard mixture containing D3-L-methionine (100 ppm, TRC, Canada), 13C9-phenylalanine (100 ppm, CIL, USA), D6-L-2-aminobutyric acid (100 ppm, TRC, Canada), D4-L-alanine (100 ppm, TRC, Canada), 13C4-L-threonine (100 ppm, CIL, USA), D3-L-aspartic acid (100 ppm, TRC, Canada), and 13C6-L-arginine (100 ppm, CIL, USA). Then, 300 μl of pre-chilled methanol/ACN extraction buffer (67/33, v/v) was added to the plasma sample, vortexed for 1 min, and incubated at −20 °C for 2 h. After centrifugation at 4000 rpm for 20 min, 300 μl of the supernatant was taken and freeze dried. The metabolites were dissolved in 150 μl of methanol/ACN buffer (50/50, v/v) and centrifuged at 4000 rpm for 30 min. The supernatants were then injected into the MS.

Metabolomics data acquisition was completed using the same spectrometry and LC and settings as used for lipidomics, except for the following parameters: the mobile phases of positive mode were (A) H2O (0.1% formic acid) and (B) methanol (0.1% formic acid). The mobile phases of negative mode were (A) H2O (10 mM NH4HCO3) and (B) methanol/H2O (95/5, v/v) (10 mM NH4HCO3). Both positive and negative models used the same gradient (min, %B): 0, 2; 1, 2; 9, 98; 12, 98; 12.1, 2; 15, 2. The temperature of the column was set at 45 °C. The MS1 range was set at 70–1050 m/z. MS2 stepped normalized collision energy was distributed to 20, 40, 60.

Raw data were searched using Compound Discoverer v3.1 (Thermo Fisher Scientific, USA) with different libraries, including our self-built BGI library containing more than 3000 metabolites with corresponding detailed mass spectrum data. After quantification, subsequent processing steps were finished by metaX, the same as for lipidomics analysis.

Lipidomics analysis
Serum samples (100 μl) were transferred to 96-well plates and mixed with 10 μl of SPLASH LipidoMixTM Internal Standard (Avanti Polar Lipids, USA). We added 300 μl of pre-chilled isopropanol (IPA) to the plasma samples, which were then vortexed for 1 min and incubated at −20 °C overnight. The samples were then centrifuged at 4000 rpm for 20 min for protein precipitation. The supernatants were then used for MS analysis.

Lipidomics analysis was performed using a Q Exactive MS (Thermo Scientific, San Jose, USA) coupled with a Waters 2D UPLC (Waters, USA). The CSH C18 column (1.7 μm, 2.1 × 100 mm, Waters, USA) was used for separation with the following elution gradient (min, %B): (A) ACN/H2O (60/40, v/v) (10 mM NH4HCO3 and 0.1% formic acid) and (B) IPA/ACN (90/10, v/v) (10 mM NH4HCO3 and 0.1% formic acid): 0, 40; 2, 43; 2.1, 50; 7, 54; 7.1, 70; 13, 99; 13.1, 40; 15, 40. The column temperature was set at 55 °C, injection was set at 5 μL, and flow rate was set at 0.35 mL/min. For HF settings, the samples were scanned twice in both positive and negative modes. The positive and negative spray voltages were set to 3.80 kV and 3.20 kV, respectively. The MS1 range was 200–2000 m/z at a resolution of 70000 with 100 ms MIT and AGC of 3e6. The top3 precursors were set to trigger MS2 scans at a resolution of 17500 with 50 ms MIT and AGC of 1E5. The MS2 stepped normalized collision energy was distributed to 15, 30, 45. The sheath gas flow rate was set at 40 and aux gas flow rate was set at 10.

Raw data were analyzed by LipidSearch v4.1 (Thermo Fisher Scientific, USA), including feature detection, identification, and alignment. The following settings were applied: tolerance of mass shift, 5 ppm; identification grade, A-D; filters, top rank; all isomer peak, FA priority, M-score, 5; c-score, 2.0. Export quantitative data from LipidSearch were analyzed using the R package metaX104, including normalization, correction of batch effects, and imputation of missing values.

For each patient in the cohort, we computed intensity for a given lipid complex class by summing the intensity of each lipid in the class. For each lipid complex class, the intensity value of each patient was further scaled by the median value of intensity from the mild patient group. We applied the Mann-Whitney U-test (multiple comparison correction with Bonferroni) to determine significant differences in the scaled intensity of each lipid complex class among severity groups.

Differential expression of proteins, metabolites, and lipids
Expression data were first adjusted using the robust linear model (RLM) for sex and age. Following RLM, the residuals were analyzed using the two-sided Mann-Whitney rank test for each group pair and P values were adjusted using Benjamini-Hochberg. Differentially expressed proteins, metabolites, and lipids were defined based on an adjusted P value < 0.05 and absolute value of fold-change > 1.5.

Clustering
Clustering was performed using the R package ‘Mfuzz’. For mRNA from whole blood, differentially expressed genes were clustered. For proteins, metabolites, and lipids from serum, all three analytes were clustered together.

Construction of gene regulatory network
ARACNe-AP105 was employed to construct gene regulatory networks (GRNs) for each group. The variability of gene expression traits was evaluated by median absolute deviation (MAD), and the top half of genes were recruited in the network. Mutual information106 was introduced to represent the strength of the regulatory relationship between TFs and target genes, and only significant pairs are retained (P < 1 × 10-8). We also executed 100 bootstraps and applied a data processing inequality tolerance filter107. The consensus network of each group was combined by statistically significant edges across all bootstrap networks (P < 0.05, Bonferroni-corrected), based on Poisson distribution. The degree was used to evaluate the centrality of genes in the network. To ensure the robustness of our remodeled GRN, we applied Chip-X Enrichment Analysis v3 (ChEA3)108 to identify TFs that target IFN and IFN receptors, with unrecognized ones eliminated.

Quantification of cell fractions from bulk RNA-seq profiles
The estimation of abundances of immune cell types in blood tissue was performed using CIBERSORTx109 based on blood RNA-seq data.

Protein interaction network construction and functional enrichment analysis
Interaction network construction and GO BP term enrichment of proteins were conducted using the STRING110 database with default parameters.

For manuscripts utilizing custom algorithms or software that are central to the research but not yet described in published literature, software must be made available to editors and reviewers. We strongly encourage code deposition in a community repository (e.g. GitHub). See the Nature Research guidelines for submitting code & software for further information.

Data

Policy information about availability of data

All manuscripts must include a data availability statement. This statement should provide the following information, where applicable:

- Accession codes, unique identifiers, or web links for publicly available datasets
- A list of figures that have associated raw data
- A description of any restrictions on data availability

Source data are provided with this paper. The data that support the findings of this study have been deposited in European Bioinformatics Institute (EMBL-EBI).
The transcriptome data have been deposited to EBI ENA with the study accession number ERP127339 (https://www.ebi.ac.uk/ena/browser/view/PRJEB43380).
The mass spectrometry proteomics data have been deposited to the EBI ProteomeXchange Consortium via the PRIDE partner repository with the dataset identifier PXD024674 (https://www.ebi.ac.uk/pride/archive/projects/PXD024674).
The metabolites and lipids data have been deposited to EBI Metabolights with the data set identifier MTBLS2542 (https://www.ebi.ac.uk/metabolights/reviewer15a9e0ef-ab10-4612-b25a-e2b6a37624c8).
The genome association data have been deposited to EBI GWAS catalog with accession numbers (GCST90014052 accessible at ftp.ebi.ac.uk:/pub/databases/gwas/summary_statistics/GCST90014001-GCST90015000/GCST90014052/GCST90014052_buildGRCh38.tsv.gz and GCST90014053 accessible at ftp.ebi.ac.uk:/pub/databases/gwas/summary_statistics/GCST90014001-GCST90015000/GCST90014053/GCST90014053_buildGRCh38.tsv.gz).
Custom scripts for data analysis in this study were present in https://github.com/DongshengChen-TY/COVID19 (DOI: 10.5281/zenodo.4624526).

Field-specific reporting

Please select the one below that is the best fit for your research. If you are not sure, read the appropriate sections before making your selection.

☒ Life sciences ☐ Behavioural & social sciences ☐ Ecological, evolutionary & environmental sciences

For a reference copy of the document with all sections, see nature.com/documents/nr-reporting-summary-flat.pdf

Life sciences study design

All studies must disclose on these points even when the disclosure is negative.

Sample size	Blood samples from 231 COVID-19 patients without selected comorbidities were collected from Tongji Hospital and Union Hospital of Huazhong University of Science and Technology, Xiangyang Central Hospital, Hubei University of Arts and Science, and Hubei Dazhong Hospital of Chinese Traditional Medicine between 19 February and 26 April 2020.
Data exclusions	Patients who are younger than 19 years old or older than 70 years old or with selected comorbidities were excluded.
Replication	Experimental replication was not attempted.
Randomization	Randomization was not performed for this study.
Blinding	Data collection and analysis were not performed blind to the conditions of the experiments.

Reporting for specific materials, systems and methods

We require information from authors about some types of materials, experimental systems and methods used in many studies. Here, indicate whether each material, system or method listed is relevant to your study. If you are not sure if a list item applies to your research, read the appropriate section before selecting a response.

Materials & experimental systems

n/a	Involved in the study	
☒	☐	Antibodies
☒	☐	Eukaryotic cell lines
☒	☐	Palaeontology and archaeology
☒	☐	Animals and other organisms
☐	☒	Human research participants
☒	☐	Clinical data
☒	☐	Dual use research of concern

Methods

n/a	Involved in the study	
☒	☐	ChIP-seq
☒	☐	Flow cytometry
☒	☐	MRI-based neuroimaging

Human research participants

Policy information about studies involving human research participants

Population characteristics	The mean age of patients was 46.7 years old (standard deviation (SD) = 13.5), and the male to female ratio was 1.12:1. All patients were diagnosed following the guidelines for COVID-19 diagnosis and treatment (Trial Version 7) released by the National Health Commission of the People's Republic of China based on the course of illness76. The patients were classified into four groups according to disease severity: i.e., critical, severe, mild, and asymptomatic. Critical disease was defined with at least one of the following conditions: (1) ARDS requiring mechanical ventilation, (2) shock, and (3) other organ failure requiring ICU admission. Severe disease was defined with at least one of the following conditions: (1) respiratory rate ≥ 30 times/min, (2) oxygen saturation ≤ 93% at resting state, (3) arterial partial pressure of oxygen (PaO2)/fraction of inspired oxygen (FiO2) ≤ 300 mmHg, (4) pulmonary imaging showing significant progression of lesions by more than 50% within 24–48 h. Mild disease was defined as patients with mild clinical symptoms but not reaching the definition of severe disease. Asymptomatic disease was defined as patients with normal body temperature and without any respiratory symptoms.
Recruitment	231 COVID-19 patients without selected comorbidities were recruited from Tongji Hospital and Union Hospital of Huazhong University of Science and Technology, Xiangyang Central Hospital, Hubei University of Arts and Science, and Hubei Dazhong Hospital of Chinese Traditional Medicine between 19 February and 26 April 2020.
Ethics oversight	This study was reviewed and approved by the Institutional Review Board of Tongji Hospital, Tongji Medical College, Huazhong University of Science and Technology, China (TJ-IRB20200405). All enrolled patients provided signed informed consent and all blood samples were collected for the rest of the standard diagnostic tests, with no additional burden to the patients.

Note that full information on the approval of the study protocol must also be provided in the manuscript.
