# Supplementary material for: The trans-omics landscape of COVID-19
Source: Nat Commun. 2021 Jul 27;12:4543. doi: 10.1038/s41467-021-24482-1 (PMC8316550; doi:10.1038/s41467-021-24482-1)
Supplement: Supplementary file 3 — Description of Additional Supplementary Files [file 41467_2021_24482_MOESM3_ESM.pdf]

Description of Additional Supplementary Files

File Name: Supplementary Data 1

Description: Baseline characteristics of enrolled patients

File Name: Supplementary Data 2

Description: Biochemical and immunological parameters associated with disease severity

File Name: Supplementary Data 3

Description: List of all analytes and differential expression

File Name: Supplementary Data 4

Description: Whole-genome sequencing and association study

File Name: Supplementary Data 5

Description: Clustering of mRNAs

File Name: Supplementary Data 6

Description: Spearman correlation coefficients of mRNA-miRNA pairs

File Name: Supplementary Data 7

Description: Spearman correlation coefficients of mRNA-lncRNA pairs

File Name: Supplementary Data 8

Description: Clustering of proteins/lipids/metabolites

File Name: Supplementary Data 9

Description: GRN based on mRNAs for each group
